# Supplementary material for: Activation of Toll-like receptor 4 by Ebola virus-shed glycoprotein is direct and requires the internal fusion loop but not glycosylation
Source: Cell Rep. 2022 Oct 25;41(4):111562. doi: 10.1016/j.celrep.2022.111562 (PMC9637988; doi:10.1016/j.celrep.2022.111562)
Supplement: Document S1. Figures S1–S5 and Tables S1 and S2 [file mmc1.pdf]

Cell Reports, Volume *41*

Supplemental information

Activation of Toll-like receptor 4 by Ebola virus-shed glycoprotein is direct and requires the internal fusion loop but not glycosylation

Michael J. Scherm, Monique Gangloff, and Nicholas J. Gay

SUPPLEMENTAL INFORMATION

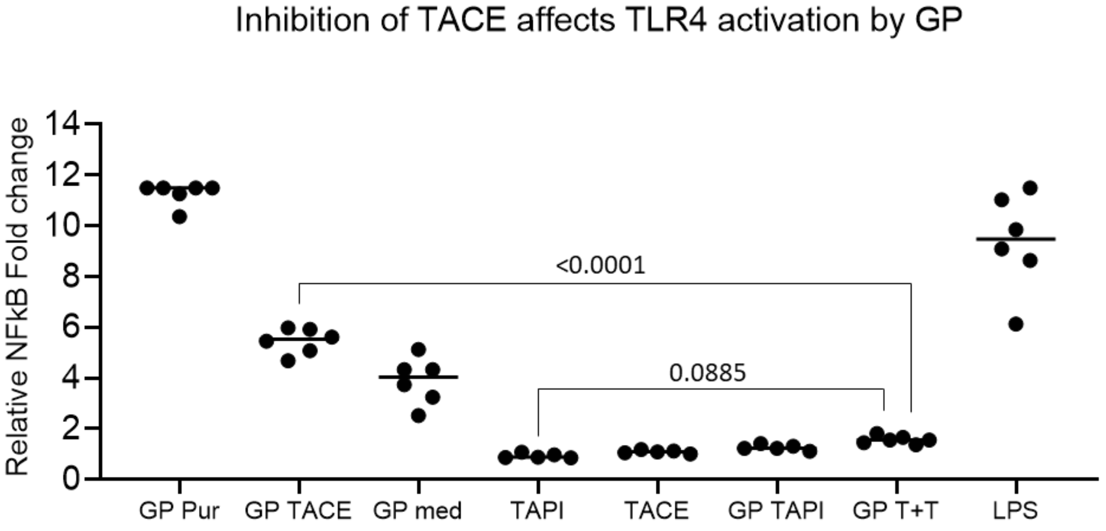

Figure S1: Inhibition of TACE eliminated GP-mediated TLR4 activation. (Related to Figure 2 and 3). The effect of inhibiting TACE activity on the secretion of GP and the activation of TLR4 was investigated. HEK293T cells were transfected with plasmids carrying full-length GP (including TMD) and/or TACE. Post-transfection induction the cells were washed and dissociated with TrypLE Express and merged with cultured Stable TMC HEK293 cells. The TACE inhibitor TAPI-1 was added to selected wells at 10 µM concentrations. The two cell lines were co-cultured for 24 hours and harvested for assessment of NFκB activity. 500 ng/µl of purified GP and 100ng/µl of LPS were used as positive controls. Expression of TACE alone, and transfection with an empty vector together with TAPI served as the negative control. The negative control was set to 1-fold change and the values normalised to it. Data points present individual replicates. Statistical significance was assessed using a one-way ANOVA test.

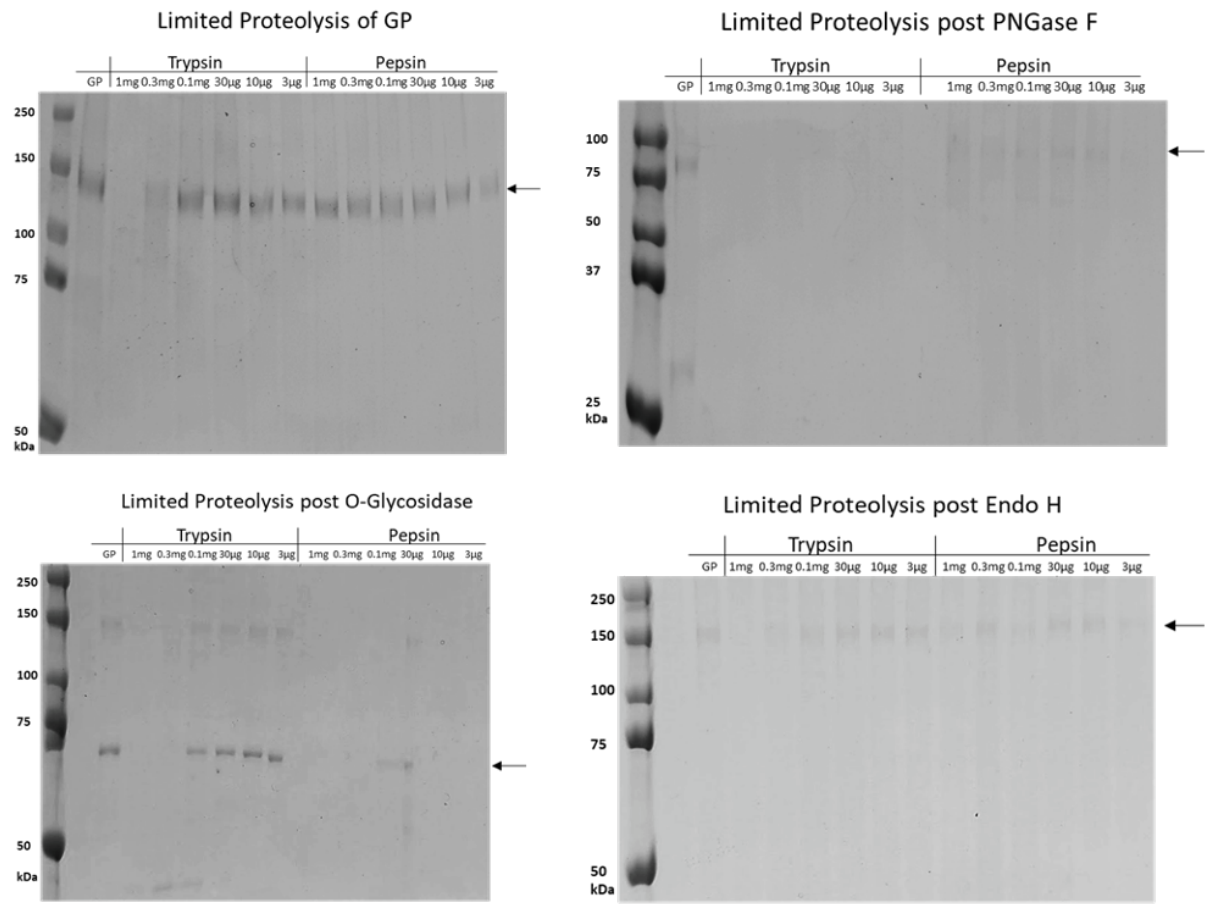

Figure S2: Partial proteolysis of GP post-deglycosylation with Trypsin and Pepsin. (Related to Figure 4). The impact of deglycosylation by PNGase F, O-glycosidase and Endo H on the stability of GP was analysed by utilising partial proteolysis by the proteases Trypsin and Pepsin, at pH 7.0 and pH 4.5 respectively. The GP samples treated with proteases under different concentrations were run on a gradient 6-16% SDS-PAGE under non-reducing conditions. The concentrations of the proteases used for each sample are indicated by the value and amount/per ml for each lane. The detailed protocol is listed in section 7. The arrow indicates GP.

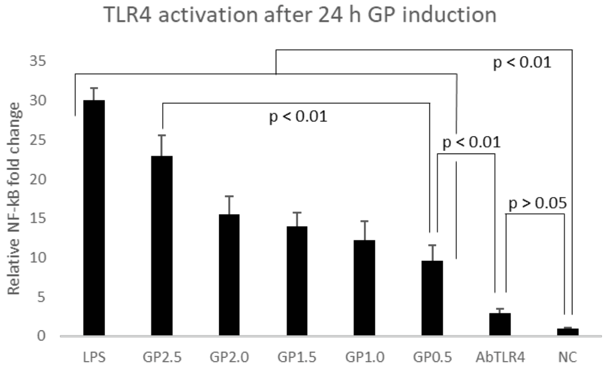

Figure S3 Dose-response curve for sGP activation of TLR4. ***(Related to Figure 5).*** NFκB activation was assayed at 5 different concentrations of sGP1,2 and in the presence of anti-TLR4 antibody.

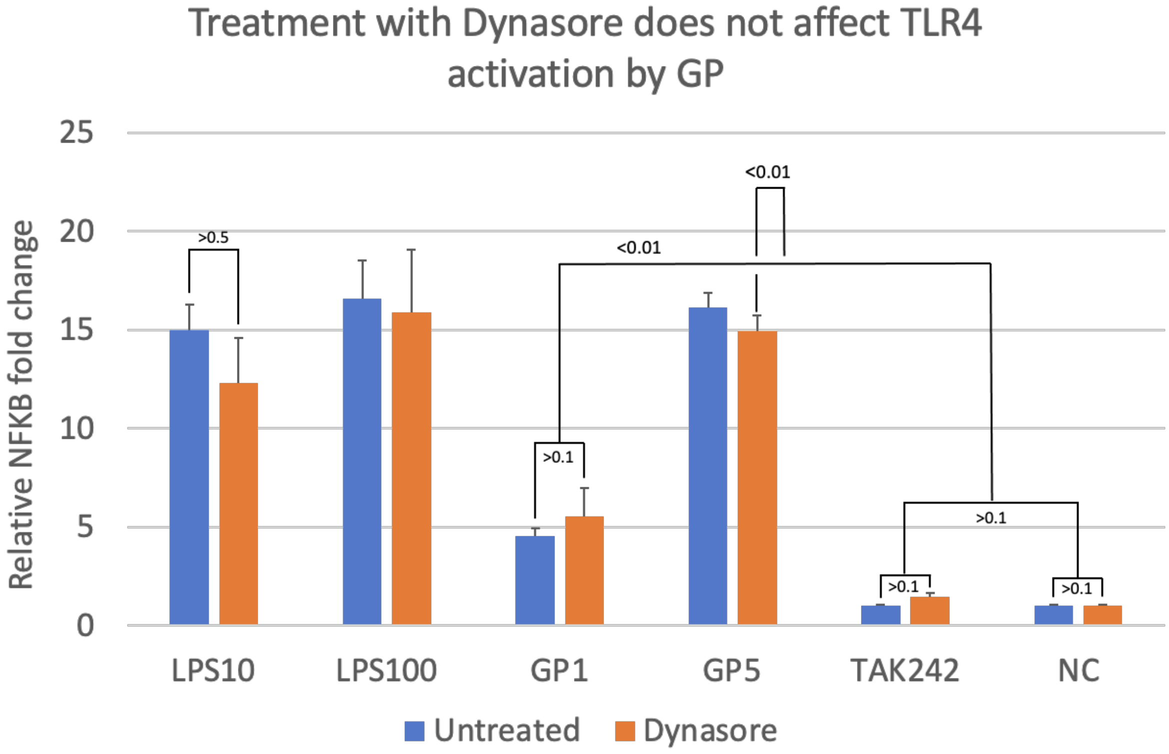

Figure S4 Treatment with Dynasore does not affect TLR4 activation by GP (related to Figure 5). Cells were either untreated (blue) or pre-treated with dynasore (80μM, orange) and then activated with the indicated ligands or the TLR4 inhibitor TAK242.

Position of Glycan	Sequence	Mutation	Expression
40	NSTL	N40D	No
40	NSTL	T42V	Yes
204	NATE	N204D	No
204	NATE	T206V	Yes
228	NETE	N228D	Yes
228	NETE	T230V	Yes
238	NLTY	N238D	Yes
238	NLTY	T240V	Yes
257	NETI	N257D	Yes
257	NETI	T259V	Yes
268	NTTG	N268D	No
268	NTTG	T270V	Yes
296	NLTR	N296D	No
296	NLTR	T298V	Yes
563	NETT	N563D	No
563	NETT	T565V	Yes
618	NITD	N618D	Yes
618	NITD	T620V	No

Table S1: Individual glycosylation site mutants of GP (related to Figure 4). Summary of the N-linked glycosylation sites of GP selected for mutant activity screen. The position and sequence of each site within the peptide chain is noted including the mutations of Asn to Glu and Thr to Val. The grey marked rows contain the mutants which did not express.

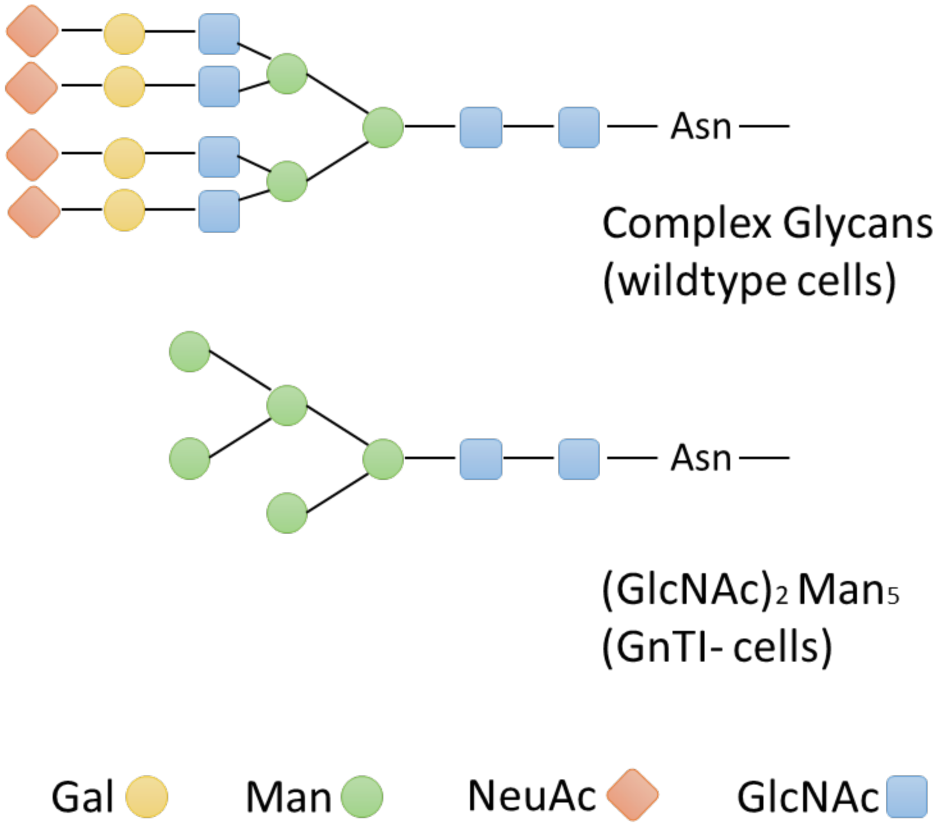

Figure S5: Schematic depiction of possible glycosylation in Expi293 GnTI- cells *(related to Figure 4 and Figure 6).* Comparison of glycosylation in HEK293T cells and GnTI- HEK cells. GnTI, N-acetylglucosaminyltransferase I; Gal, Galactose; Man, Mannose; NeuAc, N-Acetyl-Neuraminic Acid; GlcNAc, N-Acetylglucosamine; Asn, Asparagine.

Construct name	Forward primer	Reverse primer
GP pDisplay	ATATATAGGGCCCATCCCGCTTGGAGTTATC	ATTCTGTATATGCAAATTTGTCTTTTAGG GCGCGCCATATATAT
GP Synbio	GCCGCCAAGCTTGCGATCCCGCTTGGAGTTAT	GGCGGCAAGCTTCGCCTAAAAGACAAAT TTGCATATA
GP GP1	TAGGGCGCGCCGATGGTG	TCTTCGAGTCCTTCTCCCGCC
GP GP2	GAAGTAATTGTCAATGCTCAACCCAAATGCAACCCCA ATTTACATTACTGGACTACTCAG	GCTGCCGCCGCCGCCGTG
GPN40D	AGTTATCCACGATAGTACATTACAGGTTAGTGATGTC	CCAAGCGGGATGCTGCCG
GPT42V	CCACAATAGTGTATTACAGGTTAGTGATG	ATAACTCCAAGCGGGATG
GPN204D	AGAGCCGGTCGATGCAACGGAGG	CTCAAGGGGTGTGAGCTGAAG
GPT206V	GGTCAATGCAGTGGAGGACCCGTC	GGCTCTCTCAAGGGGTGT
GPN228D	TTTTGGAACTGATGAGACAGAGTAC	CCGGTAGCCTGATATCTAATTG
GPT230V	AACTAATGAGGTAGAGTACTTGTTCGAGGTTG	CCAAAACCGGTAGCCTGA
GPN238D	CGAGGTTGACGATTTGACCTACG	AACAAGTACTCTGTCTCATTAG
GPT240V	TGACAATTTGGTCTACGTCCAACTTG	ACCTCGAACAAGTACTCTG
GPN257D	GCTCCAGCTGGATGAGACAATAT	AGAAACTGTGGTGTGAATC
GPT259V	GCTGAATGAGGTAATATATGCAAGTGGG	TGGAGCAGAAACTGTGGT
GPN268D	GAAGAGGAGCGACACCACGGGAA	CCACTTGCATATATTGTCTCATTCAGC
GPT270V	GAGCAACACCGTGGGAAAACTAATTTG	CTCTTCCCACTTGCATATATTG
GPN296D	AACTAAAAAAGACCTCACTAGAAAAATTCGCAGTG	TCCCAGAAGGCCCACTCC
GPT298V	AAAAAACCTCGTTAGAAAAATTCGCAGTGAAG	TTAGTTTCCCAGAAGGCC
GPN563D	GCAGCTGGCCGACGAAACGACTC	CTCAACCCACAGATTAAACCATCTTGG
GPT565V	GGCCAACGAAGTGACTCAAGCTCTCCAAC	AGCTGCCTCAACCCACAG
GPN618D	TTGGACCAAGGACATAACAGACA	TCATGTGGTTCGATACAG
GPT620V	CAAGAACATAGTAGACAAAATTGATCAGATTATTC	GTCCAATCATGTGGTTCG
GPΔMLD	ACTCATCACCAAGATACC	TGATACAGCTGTGAAAGAC
GPΔTM	TGGTGGACAGGATGG	CACACTGGACTAGTGCCATCCTGTCCACC A
GP_MLD	ATGATGACCGGTGAACTGAGCTTCACCGT	CATCATGGTACCTGTGTTGGTGATCAGTC C
GP_485	ATGATGACCGGTATCCCCCTGGGCGTGAT	CATCATGGTACCTGTGTTGGTGATCAGTC CCAGC
GP_MLD_50 1	ATGATGACCGGTGAACTGAGCTTCACCGTGGTGT	CATCATGGTACCGCGTCTGGTTCTTCTGC CG
GP_ECD	CACGACTTCGTGGACTGAGGATCCCTGGG	GCCCAGGGATCCTCAGTCCACGAAGTCG T
GP_IFL	CTGGCCAACGAAACGACT	ATTGACAATTACTTCTCTTCGAGTC

Table S2: Primers used for EBOV GP cloning (related to Methods, Cloning and constructs). Summary of the oligonucleotides used to clone different mutants of EBOV GP. The names of each construct, the forward and the reverse primer are annotated.
