# Supplementary material for: Activation of Toll-like receptor 4 by Ebola virus-shed glycoprotein is direct and requires the internal fusion loop but not glycosylation
Source: Cell Rep. 2022 Oct 25;41(4):111562. doi: 10.1016/j.celrep.2022.111562 (PMC9637988; doi:10.1016/j.celrep.2022.111562)
Supplement: Document S2. Article plus supplemental information [file mmc2.pdf]

Cell Reports

Article

Activation of Toll-like receptor 4 by Ebola virus-shed glycoprotein is direct and requires the internal fusion loop but not glycosylation

Graphical abstract

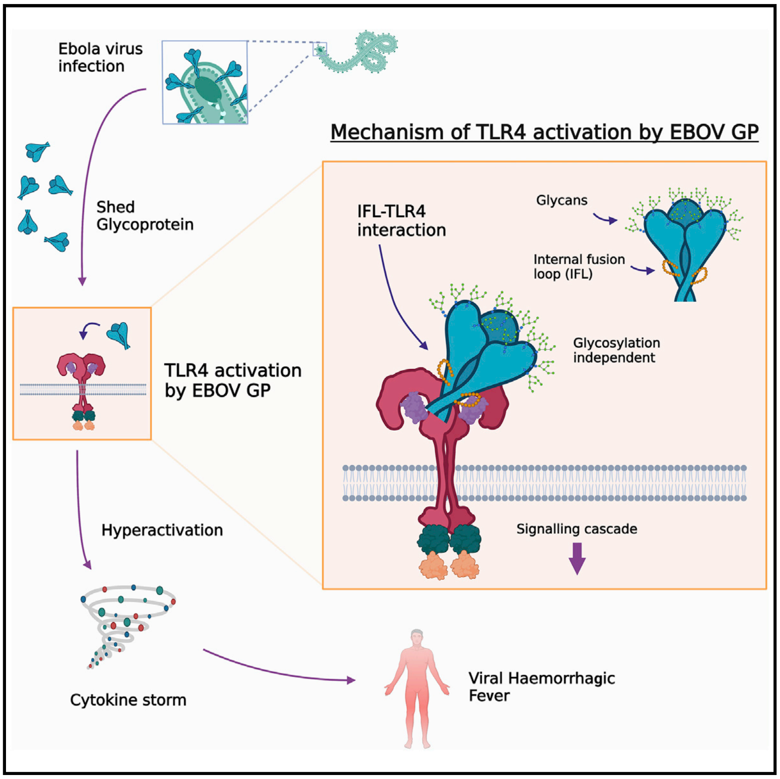

Highlights

- Glycosylation of the Ebola spike glycoprotein is not required for inflammation
- The internal fusion loop that mediates viral entry also drives inflammatory signaling
- Spike glycoprotein assembles with Toll-like receptor 4 to form an active complex

Authors

Michael J. Scherm, Monique Gangloff, Nicholas J. Gay

Correspondence

njg11@cam.ac.uk

In brief

Scherm et al. investigate how the Ebola virus spike glycoprotein (sGP) causes hyper-inflammation. They show that a hydrophobic peptide of sGP called the internal fusion loop (IFL) activates inflammatory signaling through Toll-like receptor 4 (TLR4). The IFL binds directly to both TLR4 and co-receptor MD2, forming an active signaling complex.

Scherm et al., 2022, Cell Reports *41*, 111562
October 25, 2022 © 2022 The Author(s).
https://doi.org/10.1016/j.celrep.2022.111562

CellPress

Cell Reports

CellPress
OPEN ACCESS

Article

Activation of Toll-like receptor 4 by Ebola virus-shed glycoprotein is direct and requires the internal fusion loop but not glycosylation

Michael J. Scherm,[1] Monique Gangloff,[1] and Nicholas J. Gay[1,2,*]
[1]Department of Biochemistry, University of Cambridge, Tennis Court Road, Cambridge CB2 1GA, UK
[2]Lead contact
*Correspondence: njg11@cam.ac.uk
https://doi.org/10.1016/j.celrep.2022.111562

SUMMARY

Infection by the Ebola virus, a member of the *Filoviridae* family of RNA viruses, leads to acute viral hemorrhagic fever. End-stage Ebola virus disease is characterized by a cytokine storm that causes tissue damage, vascular disintegration, and multi-organ failure. Previous studies showed that a shed form of the viral spike glycoprotein (sGP1,2) drives this hyperinflammatory response by activating Toll-like receptor 4 (TLR4). Here, we find that glycosylation is not required for activation of TLR4 by sGP1,2 and identify the internal fusion loop (IFL) as essential for inflammatory signaling. sGP1,2 competes with lipid antagonists of TLR4, and the IFL interacts directly with TLR4 and co-receptor MD2. Together, these findings indicate that sGP1,2 activates TLR4 analogously to bacterial agonist lipopolysaccharide (LPS) by binding into a hydrophobic pocket in MD2 and promoting the formation of an active heterotetramer. This conclusion is supported by docking studies that predict binding sites for sGP1,2 on TLR4 and MD2.

INTRODUCTION

Ebola virus (EBOV) is a single-stranded negative-sense RNA virus of the order *Mononegavirales* and family *Filoviridae* (Feldmann et al., 1993, 1994). It first emerged in the Democratic Republic of Congo (DRC) in 1976, and subsequently, five different species have been identified in Africa (Breman et al., 2016). EBOV is extremely dangerous, with a case fatality rate of around 50% (Leligdowicz et al., 2016). Because of this, humans are regarded as a dead-end host for the virus (Pourrut et al., 2005). The most likely natural reservoirs for EBOVs are bat species and a few non-human primates, in which the virus causes little to no pathogenicity, thus facilitating transmission (Leroy et al., 2005). Infection of humans is likely caused by spill-over events from animals, facilitating the incidence of human-to-human transmission via direct contact with bodily fluids of an infected person, with the virus being taken up through broken skin or mucous membranes in the eyes, nose, or mouth (Baseler et al., 2017).

EBOV subsequently infects and initially replicates asymptomatically in monocytes and dendritic cells, causing deregulation of both the innate and adaptive immune response (Misasi and Sullivan, 2014). The innate immune system is the first to be affected by this virus, for example by inhibition of the type I interferon response and the functional impairment of dendritic and natural killer cells (Falasca et al., 2015). After about a week, the virus becomes more widely disseminated with high levels of replication in hepatocytes and splenocytes, and this is accompanied by a massive release of cytokines, chemokines, and vasoactive substances, the so-called cytokine storm. This in turn leads to a septic shock-like syndrome termed viral hemorrhagic fever (VHF), characterized by vascular leakage and sudden hemorrhages at mucosal sites internally and externally. The excessive bleeding from the eyes and mouth, skin lesions, and bloody diarrhea rapidly deplete the patients of necessary fluids, followed by multi-system organ failure and fatal outcomes in 50% of all infections (Leligdowicz et al., 2016).

In early stages of infection, there is little production of inflammatory cytokines and chemokines because the virus effectively represses the signaling pathways of innate immunity (Misasi and Sullivan, 2014). However, this repression is overcome at the onset of severe disease, causing a dramatic increase in production of these signaling molecules by macrophages, dendritic cells, and monocytes. A landmark study (Escudero-Perez et al., 2014) showed that activation of the Toll-like receptor 4 (TLR4) inflammatory signaling pathway was responsible for the cytokine storm that is characteristic of VHF. TLR4 is a pattern-recognition receptor that is usually activated by lipopolysaccharides (LPS) from the outer membrane of Gram-negative bacteria (Bryant et al., 2010). The contribution of TLR4 to EBOV disease (EVD) fatality was subsequently supported by mouse studies, where pre-treatment or daily dosing with TLR4 antagonists including eritoran and TAK242 or anti-TLR4 antibodies increased EVD survival by up to 70% (Younan et al., 2017). Although, initially, the mechanism of EBOV activation of TLR4 was unclear, the viral glycoprotein GP1,2, a heavily glycosylated transmembrane protein, was identified as a likely activating ligand. To date, this is the only Ebola viral protein found to activate TLR4 signaling (see Figure 1 for an overview).

This is an open access article under the CC BY license (http://creativecommons.org/licenses/by/4.0/).

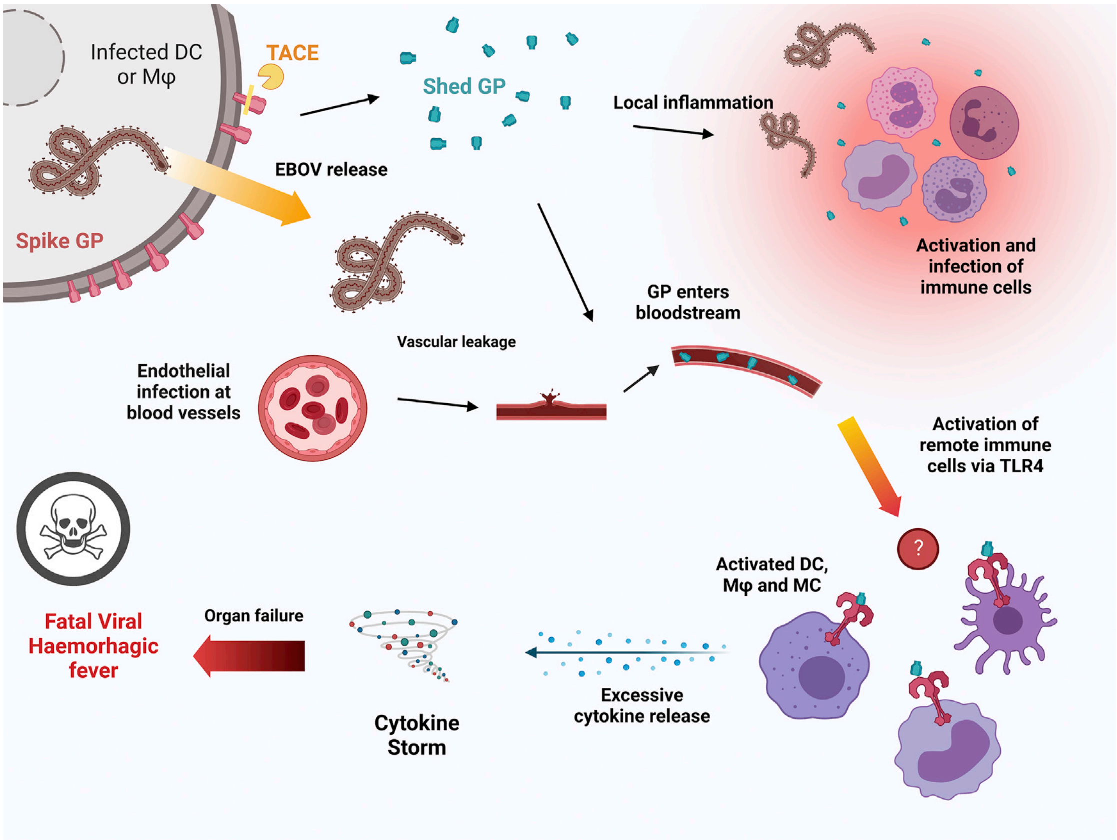

Figure 1. Shedding of GP leads to a cascade of inflammatory reactions mediated by TLR4
The sequential effects of EBOV infection and the role of GP on the host's body and immune system are depicted. Following infection, the virus replicates without activating innate immunity. Viral particles are released along with the spike GP, a target for endogenous TACE. After cleavage, shed GP (blue) activates TLR4, leading to local inflammation. The inflammatory reaction causes vascular leakage induced by endothelial cell infection. GP diffuses into the bloodstream, migrates, and activates remote cells from the original site of infection. This system-wide activation leads to the escalating activation of cytokines, formation of a cytokine storm, and finally to often fatal hemorrhagic fever and multiple organ failure.

The Ebola genome encodes seven genes: the nucleoprotein (NP), virion protein 24 (VP24), VP30, VP35, VP40, polymerase (L), and glycoprotein (GP) (Feldmann et al., 1993). Taken together in a structural context, NP, the minor NP (VP30), VP35, and the RNA L build the nucleocapsid containing the genomic RNA. VP24 and VP40 form a matrix and connect the nucleocapsid with the viral envelope, which is comprised of a lipid bilayer densely covered by the GP (Lee and Saphire, 2009). The membrane-bound trimeric GP is critical in the EBOV life cycle, as it is solely responsible for attachment, membrane fusion, and infection of host cells. Due to reading errors of the GP gene during transcription and multiple post-translational processes, three GP protein variants are expressed by the virus. Thus, a sequence of seven uracil residues within the GP gene causes transcriptional stuttering and subsequent frameshift mutation in approximately 20% of GP transcripts (Weissenhorn et al., 1998), and this gives rise to the synthesis of the 676-amino-acid-long, full-length type I transmembrane GP (pre-GP1,2). The pre-GP protein is further cleaved by Furin in the Golgi into two monomers GP1 and GP2, which finally form the "mature" heterodimer, GP1,2, through a disulphide linkage between Cys53 of GP1 and Cys609 of GP2 (Jeffers et al., 2002). The final GP1,2 assembles into a 450-kDa spike trimer at the surface. By contrast, most GP transcripts do not have a frameshift and give rise to a shorter secreted dimeric pre-shed GP (sGP) of unknown function.

Cleavage of GP1,2 by the metalloprotease tumor necrosis factor α (TNF-α)-converting enzyme (TACE) at the transmembrane domain releases a truncated GP1,2, termed sGP1,2, into the circulation (Gooz, 2010). GP1,2 is heavily glycosylated, especially the 150-residue mucin-like domain (MLD), with up to 80 sites of O- and N-linked glycosylation (Jeffers et al., 2002). The MLD is essential for the activity of the GP in causing dysregulated inflammation and cytokine storms that contribute to viral pathogenicity (Okumura et al., 2010). sGP1,2 levels are low at early stages of infection but increase with the onset of severe disease, inducing nuclear factor κB (NF-κB) and inflammatory cytokine production in uninfected macrophages and dendritic cells. Pretreatment of cells with anti-TLR4 antibodies decreases immune cell activation by sGP (Escudero-Perez et al., 2014), as does extensive deglycosylation, pointing to a direct or indirect role for N- or O-linked glycans.

CellPress
OPEN ACCESS

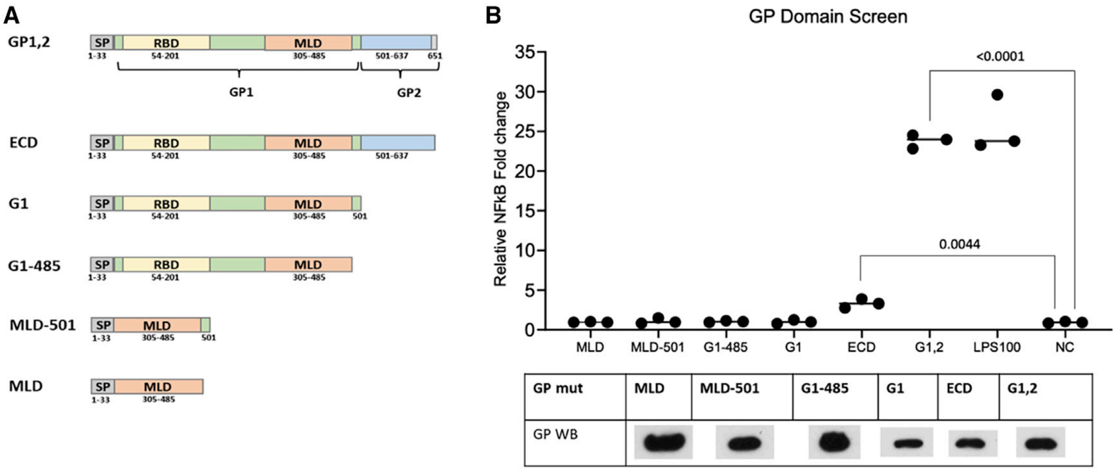

Figure 2. Truncations of GP are incapable of activating TLR4

(A) The domain boundaries are indicated by the residue position number within the polypeptide chain. The individual domains are indicated through color separation: GP1 (green), GP2 (blue), GP, glycoprotein; ECD, ectodomain; SP, signaling peptide (gray); RBD, receptor-binding domain (yellow); MLD, mucin-like domain (orange); and the C-terminal transmembrane domain starting from residue 638.

(B) GP truncation constructs expressed in HEK293T cells were tested for TLR4 activity. An NF-κB luciferase activity screen was conducted with stable TMC HEK293 cells and 5 μg/μL cell extracts of the GP truncation constructs.

The negative control consisted of medium and cell lysate from untransfected cells. The negative control was set to 1-fold change and the values normalized to it. Expression levels were verified using western blot using 5 μg protein and detected with anti-His tag antibody. Data points represent means of triplicates. Statistical significance was assessed using a one-way ANOVA test.

In this article, we identify a short hydrophobic sequence in sGP, the internal fusion loop (IFL) peptide that is necessary for inflammatory activation through TLR4. Extensive mutagenesis of glycosylation sites in sGP failed to identify specific glycans that are required for TLR4 signaling.

RESULTS

The minimal domain required for activation of TLR4 lies at the C terminus of the sGP

The domains required for activation of TLR4 were investigated by designing and cloning truncation mutants of GP. The minimal domain screen was purposely limited to the glycosylated and MLD carrying GP1, as the membrane associated GP2 is likely to be sterically hindered from interaction with TLR4 while immobilized on the virion membrane, as suggested by the binding epitopes of neutralizing antibodies (Davidson et al., 2015). Overall, five GP truncation constructs were created with pDisplay as a backbone (see Figure 2A), and their ability to activate TLR4 was compared with that of the optimized GP constructs GP1,2 (C-terminal helix of the transmembrane domain [TMD]). The first construct was created by removing the intracellular domain and TMD of GP, named the ectodomain (ECD; GP). The second construct, named GP1, was created by prematurely terminating GP1,2 at the Furin cleavage site after amino acid residue 501. The next construct shortened GP1 to only include all residues upstream and the MLD, GP-485. The last two constructs were created by only cloning MLD downstream of the signal peptide for the first and including the final few residues of GP1 in the construct MLD-501. A schematic depiction is shown in Figure 2. The GP truncation constructs were expressed in HEK293T cells and protein extracts prepared as described. NF-κB luciferase assays were then carried out using stable HEK293 cells that express TLR4, myeloid differentiation factor 2 (MD2), and CD14 (TMC) using 50 μg protein extract. GP1,2 activated TLR4 to a similar extent as LPS treatment and the ECD construct at a lower, but statistically significant, level. None of the other constructs were able to induce NF-κB in this assay, indicating that the ability of GP to induce inflammatory signaling lies in the C-terminal 150 residues.

Inhibitors of TACE prevent shedding of GP and paracrine activation of TLR4/MD2

During an active EBOV infection, GP is shed from both cells and virions due to cleavage by endogenous TACE. These sGP molecules diffuse through the blood stream and can activate TLR4 on cells remote from the infection site. Theoretically, inhibition of TACE could restrict the distribution of GP in the host, limit the TLR4 activation to the site of infection, and significantly reduce cytokine and chemokine activation. TACE or ADAM17 inhibitors have been used in clinical trials for cancer treatment and are commercially available (Moss and Minond, 2017).

To investigate this hypothesis, a cell-based co-culture and inhibition assay was created. HEK293T cells were transfected with a full-length GP plasmid that includes the TMD. Additionally, a representative sample of cells were transfected with TACE-containing plasmids. In parallel, stable HEK293 cells expressing TLR4, MD2, and CD14 were cultured and prepared for NF-κB signaling assays in 96-well plates. Twenty-four h post-transfection, the GP-expressing cells were washed and dissociated with TrypLE Express, and the stable cell culture and the GP-expressing cultures were merged. The medium of selected wells was supplemented with 10 μM TAPI-1, a TACE specific inhibitor. The two cell lines were co-cultured for 24 h and assayed for NF-κB signaling assays (Figure S1). A control with purified GP

was included in the co-culture for signal strength comparison. Purified GP induced NF-κB by about 10-fold, about the same as LPS. The samples with co-cultured cells expressing GP alone showed up to 5-fold induction of NF-κB, and co-expression with TACE yielded up to 6-fold induction, although this was not statistically significant. As expected, the treatment with TACE inhibitor TAPI-1 eliminated GP-mediated NF-κB activation even when TACE was co-expressed.

Activation of TLR4 by sGP1,2 does not require discrete N- or O-linked glycosylations

To assess the impact of glycosylation on the activation of TLR4, the protein sequence of sGP was analyzed with the glycosylation prediction software NetNGlyc and NetOGlyc (Gupta and Brunak, 2002), which indicates the probability of a glycosylation occurring *in vivo*. The software predicted 17 possible N-linked glycosylation sites, 15 for GP1 and two for GP2. Ten of these showed a high glycosylation potential (>0.5) and were enriched in the glycan cap (residues 200–300) at positions 40, 204, 228, 238, 257, and 268, while the others were predicted to be at positions 317, 386, 436, and 563 located within or close to the MLD. Of over 80 predicted O-linked glycosylation sites, five were predicted to have a high likelihood, most of which were located at the MLD (data not shown). The theoretical molecular weight of all 97 predicted glycans is 75 kD, and the sGP polypeptide chain is also about 75 kDa. This is consistent with the apparent molecular weight of sGP estimated by SDS-PAGE of GP of 140–150 kDa.

Based on the predicted N-glycosylation sites, GP mutants were designed with individual altered glycosylation sites. The chosen mutations were located outside of the unstructured MLD: seven in the GP1 N-terminal glycan cap domain (40, 204, 228, 238, 253, 268, and 296) and two within the C-terminal region of GP2 (563 and 618) (Table S1). The MLD glycosylation sites were deemed unlikely to have a role in the activation of TLR4 but rather to function in immune evasion and epitope shielding. The N-glycosylation sequons of Asn-Xaa-Ser/Thr were mutated to Gln-Xaa-Ser/Thr or Asn-Xaa-Val to prevent the addition of glycans. In some cases, double mutants were generated because a previous study found that at some single sites, mutants of N to Q or S/T to V caused reduced expression and protein instability (Lee and Saphire, 2009). Of the 18 glycosylation site mutants, 12 expressed in HEK293T cells (Figure 3A; Table S1) representing mutants of all nine sites selected. Mutant GPs were tested for the ability to activate signaling using an NF-κB reporter assay. All mutant GPs activated TLR4 in this assay, and no significant reduction in activity was observed for any mutant, compared with the wild-type (WT) GP (Figure 3B).

The results above might be explained if multiple N- or O-linked glycans are needed to activate TLR4. We therefore used enzymes to produce globally deglycosylated sGP. O-glycosidase was used to remove O-linked glycans, of which over 80 are predicted to cover the MLD of GP. To assess any roles of N-linked glycans, PNGase F, Endo H, galactosidase, and neuraminidase were used in the de-glycosylation assay (see Figure 3C for enzyme specificities). In TLR4 reporter assays, the GP samples treated with O-glycosidase and PNGase F showed a significant reduction in NF-κB activation compared with untreated GP and baseline activation at the level of the negative control (Figure 3D). By contrast, the samples treated with galactosidase and neuraminidase did not show a significant change compared with untreated GP. Surprisingly, the Endo H-treated sample did not show a significant reduction in activity, although the rate of glycan removal is comparable to PNGase F.

In further studies, GP lacking complex glycans was purified from the Expi293F GnTI, a cell line that does not express N-acetylglucosaminyltransferase I (GnTI) (Figure 3E). The ability of purified GNTI− GP to activate TLR4 was compared with HEK293T-derived GP using a NF-κB signaling assay. The difference between the two protein samples was not significant (Figure 3F). A change in glycosylation from complex glycans to simple mannose glycosylation did not affect the ability of GP to activate TLR4.

Following the observation that the complete removal of both N- and O-linked glycans abolished TLR4-induced NF-κB activity, the samples were further analyzed to assess why the absence of glycans may influence GP's ability to activate TLR4. One explanation was that complete glycan removal may impact GP stability, in turn preventing the TLR4 activation seen following O-glycosidase and PNGase F incubation. To investigate this hypothesis, partial proteolysis was carried out (Figure S2). The complete removal of glycans, both N- and O-linked, makes GP more susceptible to proteolytic degradation, with the lack of N-glycans making the protein more susceptible to trypsin, while the lack of O-glycosylation significantly increases degradation by pepsin.

GP activates TLR4 with a linear dose response and competes with the *Rhodobacter spheroides* lipid A antagonist

To investigate whether the activation of TLR4 by sGP1,2 follows a linear dose response similar to LPS (Park et al., 2009; Prohinar et al., 2007), stable TMC HEK293 cells were incubated with a varying amount of GP. In this assay, the cells plated in 96-well plates were subjected to GP protein ranging from 0.5 to 2.5 μg (0.035–0.17 μM) and 100 ng/μL LPS (6.5 μM) for 24 h. Incubation with the lowest amount of GP led to a 10-fold increase in NF-κB signaling, while the highest amount of GP increased up to 25-fold (Figure S3) and followed a linear progression. The presence of anti-TLR4 antibody in the assay results in a significant reduction in activity, confirming that the NF-κB activity is mediated by TLR4. The observed dose response is consistent with the progressive cytokine activation and cytokine storm observed in late-phase Ebola disease.

To further investigate the mechanism by which EBOV GP activates TLR4, we used an antagonist isolated from *R. spheroides* (RSLA) that binds to the hydrophobic pocket of MD2 but does not activate signaling and competes with agonistic LPS.

To investigate this, cells were pre-treated with three different concentrations of RSLA (100, 300, or 1,000 ng/μL) 1 h prior to a 6-h induction with either LPS or GP at two different concentrations each, 10 and 100 ng/μL and 100 and 500 ng/μL, respectively. Signaling activity was measured with an NF-κB assay and assessed relative to untreated controls. In all cases, pretreatment with RSLA inhibited signaling induced by LPS and GP, indicating that both molecules compete with RSLA for binding in the hydrophobic pocket of MD2 (Figure 4A).

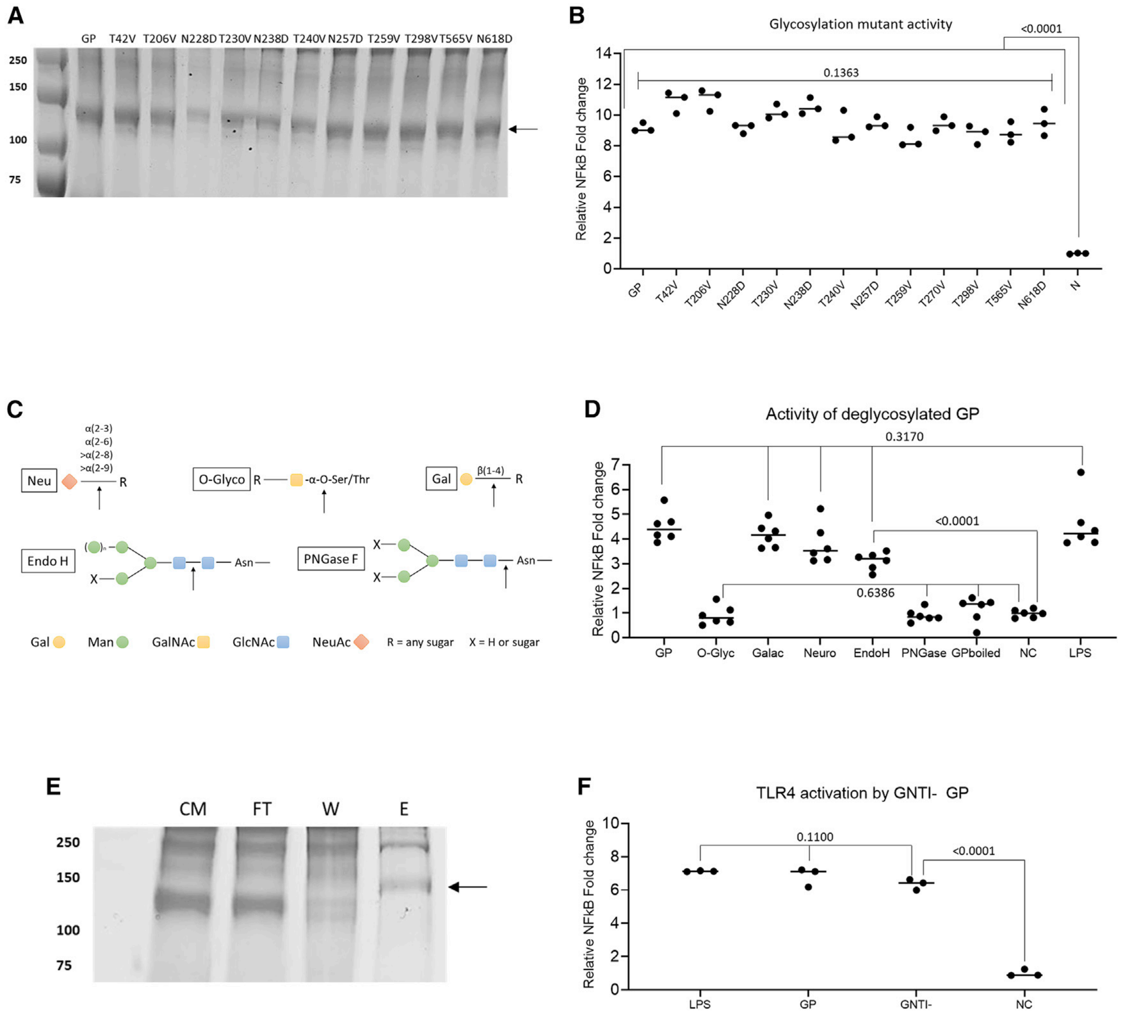

Figure 3. Individual glycosylation site mutants of GP do not affect TLR4 activity, whereas complete removal of glycans does

(A and B) GP with single glycosylation site mutants (Table S1) were expressed in HEK293T and purified (A), and their ability to activate TLR4 was measured with an NF-κB reporter assay (B).

(C and D) WT GP was treated with de-glycosylation enzymes (C), and their TLR4 activation capabilities were assessed with NF-κB signaling assays (D).

(E and F) GP was expressed in Expi293F GnTI− cells and purified (black arrow: purified GP; CM, cell medium; FT, flow through; W, wash fraction; E, elution) (E), and TLR4 activity investigated with NF-κB signaling assays (F).

For (B), (D), and (F), 1 μg/μL GP and 100 ng/μL LPS were used. The negative control consisted of purified GP, buffer exchanged through a polymyxin B column to remove LPS, treated with proteases for 1 h, and followed by boiling for 20 min. The negative control was set to 1-fold change and the values normalized to it. Data points present individual replicates (B and D) and means of triplicates (F). Statistical significance was assessed using a one-way ANOVA test.

In light of this, we analyzed GP for hydrophobic regions using the Expasy Protscale software (Figure 4B), particularly those located in regions that may be accessible through a conformational change. Of the hydrophobic regions identified, the signaling peptide and the TMD were disregarded due to the signaling peptide being cleaved in the native protein and the TMD being inaccessible due to immobilization within the cell or virion membrane. The putative receptor-binding domain would also be inaccessible as it is located in the core of the trimeric spike GP. Hit 3 is located at the peak of the propellor-like MLD, and it would be natively shielded by glycans, also making it inaccessible. By contrast, hit 4, which is part of the IFL required for membrane fusion and cell infection (Lee et al., 2008; Lee and Saphire, 2009), is situated roughly in the center of the spike protein at the stalk. The flexible nature of the loop and the accessible location lacking glycans or other shielding domains make the IFL the most likely motif to interact and activate the TLR4/MD2 complex.

The IFL of GP is required for activation of TLR/MD2 signaling

To further explore this hypothesis, a mutant of GP was created lacking the hydrophobic part of the IFL (deleted hydrophobic region of fusion loop in bold typeface), 524-GAA**IGLAWIPYFGP**AA-539, termed GPΔFuLo (see Figure 6D and 6E). The GPΔFuLo mutant was successfully expressed and purified with the same procedure as for WT sGP1,2. Once

A

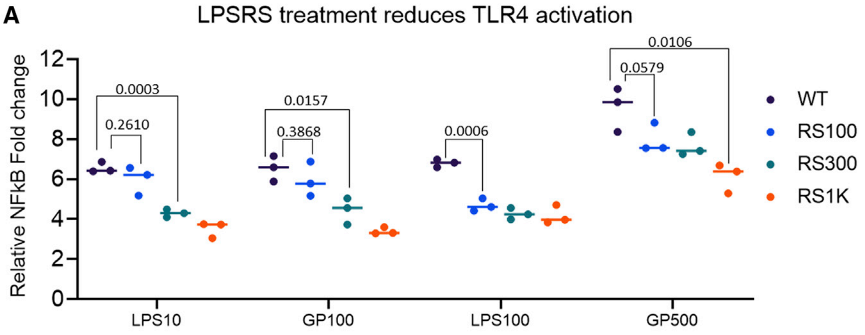

B

No	Residues	Region/Domain
1	20-29	Signalling peptide
2	177-187	Receptor binding domain
3	478-493	C-terminus of MLD
4	527-535	Internal fusion loop
5	654-672	Transmembrane domain

C

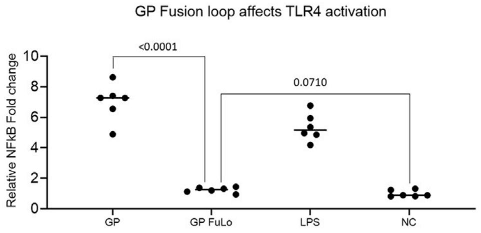

Figure 4. The internal fusion loop of GP is critical for TLR4 activation

(A) The pre-treatment of stable hTLR4/MD2/CD14 HEK293 cells with RSLA reduces TLR4 activation by GP and LPS equally, suggesting a similar mechanism. The cells were pre-incubated with RSLA at concentrations of 100 ng/μL, 300 ng/μL, and 1 μg/μL for 1 h pre-induction, followed by LPS concentrations of 10 and 100 ng/μL and 100 and 500 ng/μL of GP for 6 h, and NF-κB activity was assessed.

(B) Sequence analysis of GP according to the Kyte and Doolittle scale for hydrophobicity reveals five regions of high hydrophobicity.

(C) Deletion of the internal fusion loop sequence, hit no. 4, in fully glycosylated GP diminished TLR4 activation as evident from NF-κB signaling assays. The cells were incubated with 100 ng/μL LPS and 500 ng/μL GP for 6 h, and activity was assessed. The negative control consisted of purified GP, treated with proteases for 1 h, and was followed by boiling for 20 min.

The negative control was set to 1-fold change and the values normalized to it. Statistical significance was assessed using a one-way ANOVA test. Data presented as means of triplicates (A and C).

purified, 200 ng/μL GP protein was incubated with stable TMC HEK293 cells, and the relative levels of NF-κB signaling were compared with WT GP (Figure 4C). WT sGP1,2 induced a 7-fold increase in signaling, but GPΔFuLo showed no significant change to the normalized negative control and was deemed inactive.

GP, but not the fusion loop mutant, binds directly to MD2 and TLR4

To further investigate the role of the IFL, we carried out co-immunoprecipitations with purified TLR4 and MD2 with WT sGP1,2 and GPΔFuLo. Protein deglycosylated with ENDO H, PNGase F, O-glycosidase, and GP expressed in HEK293 GNTI− cells were also tested (Figures 5 and S5). Immunoprecipitation revealed binding of GP to MD2 and TLR4 as indicated by the visible band of around 150 kDa. Despite the difference in TLR4 activation observed after deglycosylation, all GP glycosylation samples, including GP derived from GNTI− were able to bind both TLR4 and MD2, as indicated by bands at 150 kDa for GNTI− GP and ENDO H-treated GP, 75 kDa for PNGase F-treated GP, and between 50 and 75 kDa for O-glycosidase-treated GP. By contrast GPΔFuLo, the fusion loop mutant, did not bind to MD2 or to TLR4. These results are consistent with the signaling experiments (Figure 4C), except that extensively deglycosylated samples that were unable to signal still formed complexes with TLR4/MD2. This indicates that the inhibition of signaling observed is an indirect effect perhaps due to defects in protein folding or instability.

Protein-protein docking predicts binding sites for sGP1,2 and TLR4/MD2

To further investigate whether GP activates TLR4 signaling by an analogous mechanism, we carried out a series of docking simulations using GRAMM-X (Tovchigrechko and Vakser, 2006) and HDOCK (Yan et al., 2020) with the activated TLR4/MD2 heterotetramer as receptor and the fusion loop or monomer sGP1/2 as ligand. Strikingly, the fusion loop peptide alone is strongly predicted to bind in the hydrophobic binding pocket of MD2 (Figure 6A) in a configuration that is nearly identical to that of ligand LPS (Figure 6B). In full-length sGP1,2 trimer, the IFL is sequestered by binding to an adjacent subunit but is released upon dissociation into a monomer, allowing binding to MD2 (Figures 6C–6F). A close-up view shows the IFL adopting a compact hydrophobic configuration similar to that see in previous nuclear magnetic resonance (NMR) studies (Figure 6G) (Gregory et al., 2011).

Protein-protein contacts involving sGP1,2 and residues from both TLR4 chains and MD2 are also observed (Figure 7A). Two copies of Ebola GP1/2 GPs are predicted to crosslink TLR4/MD2 into a signaling-competent complex (Figure 7B). In addition, sGP1/2 displays additional contacts outside the hydrophobic cavity of MD2, to which the IFL binds, increasing the interface to also potentially include glycan-mediated contacts (Figure 7C).

DISCUSSION

Previous studies presented evidence that the glycans of GP are necessary for the activation of TLR4, as deglycosylation caused significant reduction in signaling activity (Escudero-Perez et al., 2014). In this study, the global removal of GP glycans does cause reduced TLR4 activation, but our results suggest that this is most likely due to instability or degradation of GP in culture, as the interaction between TLR4 and MD2 remains unaffected (Figure 5). It remains possible that two N-linked glycosylations at

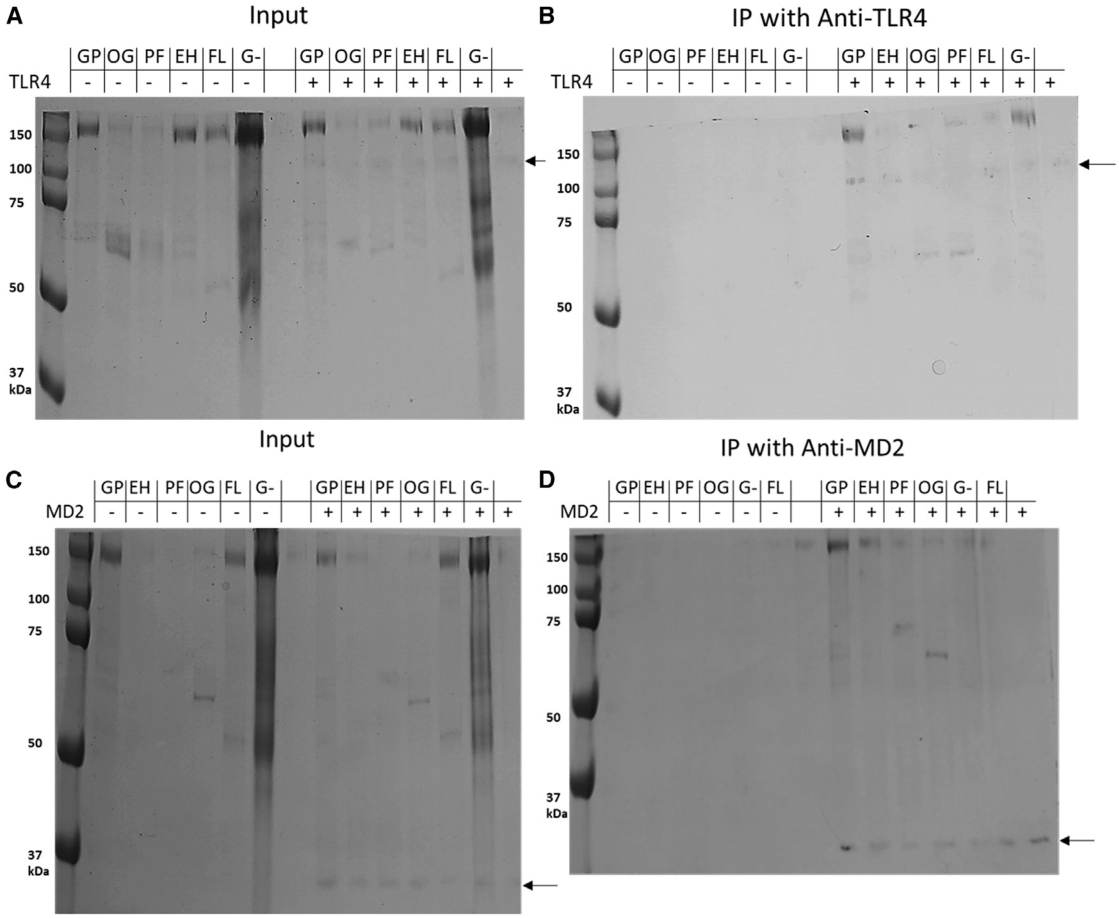

Figure 5. WT and deglycosylated GP interacts directly with TLR4 and MD2 except for the fusion loop mutant GPΔFuLo

Immunoprecipitation conducted with purified TLR4, MD2, and GP with different states of glycosylation via specific antibodies for the human proteins. Here, 2 μg TLR4 and MD2 and 5 μg GP were incubated and immunoprecipitated with either anti-TLR4 or anti-MD2 antibodies immobilized to protein A/G beads.

(A) Input for co-immunoprecipitation (coIP) with TLR4.

(B) All GP species were co-precipitated with TLR4 except GPΔFuLo.

(C) Input for coIP with MD2.

(D) All GP species were co-precipitated with MD2 except GPΔFuLo. All deglycosylated GP species were capable of binding TLR4. The "+" indicates the presence of TLR4 or MD2 whereas the "–" indicates the absence in the coIP sample.

Black arrows indicate TLR4 (A and B) and MD2 (C and D). GP, WT glycoprotein; OG, GP protein after treatment with O-glycosidase; PF, GP after treatment with PNGase F; EH, GP after treatment with Endo H; FL, GP with deleted internal fusion loop GPΔFuLo; G−, GP expressed in Expi293 GnTI− cells.

N563 and N618 may have a role in TLR4 activation. These glycans are critical for trimer assembly and post-translational modifications, including de-mannosylation (Wang et al., 2017). Accordingly, mutant protein is unstable and difficult to express. However, given the evidence of a primary role for the IFL presented, it is unlikely that these glycans have a direct function in TLR4 signaling.

By contrast, a mutant GP lacking the IFL is unable to activate or interact with TLR4/MD2. These results provide compelling evidence that direct interaction of the IFL with TLR4 and MD2 is the cause of inflammatory signaling in EVD. Structural analysis of TLR4/MD2 reveals that the acyl chains of bacterial ligand LPS bind into a hydrophobic pocket of MD2 and induces the formation of an active heterotetramer that is stabilized by a network of hydrophobic and electrostatic interactions with MD2 and the ECD solenoid (Park et al., 2009). Interestingly in addition to LPS, a number of structurally diverse hydrophobic molecules including cardiolipin (Pizzuto et al., 2019), paclitaxel (Zimmer et al., 2008), the synthetic peptidomimetic neoseptin 3 (Wang et al., 2016b), and oxidized phospholipids (Mancek-Keber et al., 2015) are also able to bind MD2 acting as both inhibitors and activators. On the other hand, two agonists, the cationic lipid C14 amidine (Lonez et al., 2015) and nickel ions (Raghavan et al., 2012), bind directly to TLR4 ECD, stabilizing the homo-dimerization interface required for signaling. A further class of endogenous activators such as the chromatin protein HMGB1 and S100A8 may have an indirect mode of action, sequestering hydrophobic activators and presenting them to TLR4/MD2 (Bryant et al., 2015).

Our docking analysis strongly predicts that the IFL binds to the MD2 hydrophobic pocket and that monomeric sGP1/2 promotes

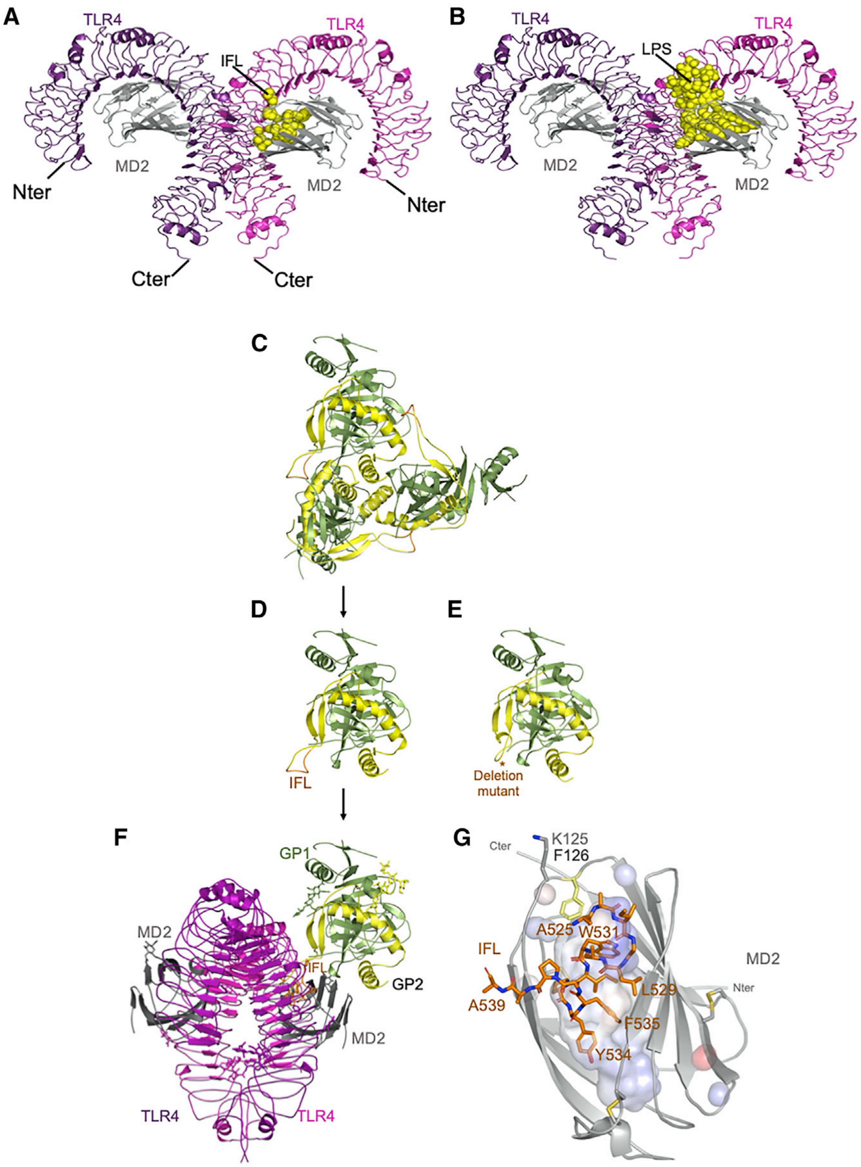

Figure 6. The IFL of Ebola glycoprotein GP1/2 docks in the LPS binding site of the TLR4/MD2 dimer

(A and B) The best docking pose for IFL (A) overlaps perfectly with LPS binding site of MD2 (B). Both are depicted in yellow spheres.

(C) Trimeric prefusion GP1,2 as observed in crystal structure 3CSY (Lee et al., 2008).

(D) Monomeric sGP1, 2 in the same orientation as observed in the trimer.

(E) IFL deletion mutant (marked with asterisk) maintains the overall fold of GP1/2 but is unable to crosslink TLR4/MD2 into a signaling competent complex.

(F) TLR4-MD2 can be crosslinked by one or two sGP1,2 molecules (second binding site not represented).

(G) Close-up view of the IFL inside MD2 hydrophobic cavity. Structural representations are generated with The PyMOL Molecular Graphics System, v.2.5.2, Schrödinger.

formation of an active heterotetramer (Figure 7B). It is unlikely that trimeric GP1/2 would be able to bind with TLR4 or MD2 due to steric hindrance. However, it is known that sGP1/2 is metastable and that the shed form is present in the circulation and culture supernatants as both monomers and trimers (Dolnik et al., 2004; Rutten et al., 2020). The docking analysis is consistent with both signaling and interaction analyses. In future work, we will investigate specific TLR4 and MD2 mutants that effect responses to LPS such as F126 in MD2 the “gatekeeper” residue to the hydrophobic core (Bryant et al., 2010). Indeed, F126 (shown in yellow in Figure 6G) is close to IFL residues Ala525 and Trp531 in the docked structure.

The principal function of the IFL is the fusion of the viral and endosomal membranes during infection leading to release of viral RNA into the cytosol of the cell. In the pre-fusion conformation of GP, the hydrophobic side chains of IFL are sequestered by binding to a region of an adjacent GP1 monomer (Lee et al., 2008) but undergo a conformational change in the endosome, enabling the IFL to insert into the target host membrane. This general mechanism is shared with a number of other viruses including influenza (Olejnik et al., 2018) and it may be that these IFLs can also activate TLR4. Interestingly, Dengue virus lacks a fusion loop but contains exposed hydrophobic domains in non-structural protein 1 (NS1) that mediate membrane interactions and are likely to play a role in TLR4 activation (Akey et al., 2014; Modhiran et al., 2015).

During an infection by EBOV, the levels of GP may reach concentrations higher than 34 μg/mL in the blood (Escudero-Perez et al., 2014), as estimated from animal studies with infected guinea pigs. In our dose-response curve (Figure S3), a GP concentration of 25 μg/mL (0.17 μM) gave a similar response as 100 ng/mL (6.6 μM) of LPS, indicating that TLR4 activation by sGP1/2 is in the physiological range. During infection, the full-length form of GP1,2, including the TMD, is expressed, incorporated into newly formed virions, and presented at the cell membrane of infected cells. The high levels of sGP1/2 in the blood lead to activation of targets and secretion of cytokines remote from the site of infection due to the cleavage of GP1,2 into sGP1/2 by the endogenous TACE/ADAM17. Although inhibition of TACE should lead to the reduction of sGP, keeping GP immobilized at the membrane, a therapeutic role for TACE inhibitors has not been explored. Here, we show that the inhibition of TACE/ADAM-17 reduces the amount of sGP and that the activation of TLR4 is almost eliminated. In this regard, the Reston EBOV (RESTB), which is non-pathogenic to humans, does not strongly activate the TLR4 inflammatory immune response in

CellPress
OPEN ACCESS

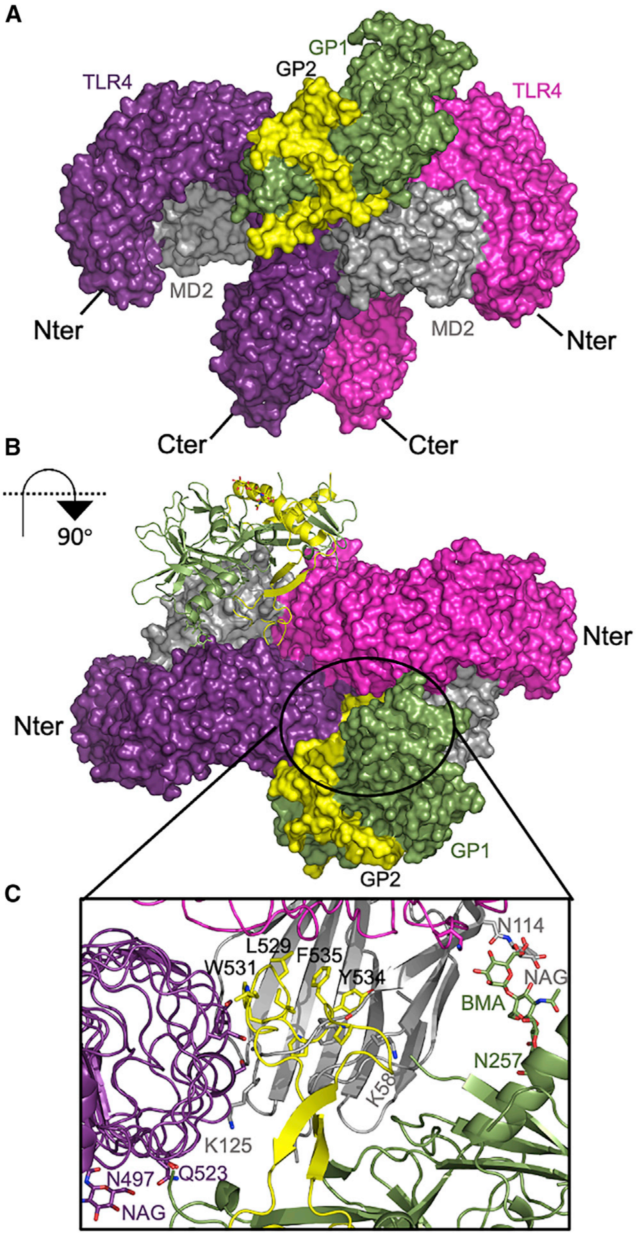

Figure 7. sGP1,2 binds TLR4/MD2 beyond the LPS binding pocket
(A) Protein-protein contacts include residues from both TLR4 chains as well as MD2.
(B) Two copies of Ebola GP1/2 glycoproteins crosslink TLR4/MD2 into a signaling competent complex.
(C) GP1/2 displays additional contacts outside the hydrophobic cavity of MD2, to which the IFL binds, increasing the interface to also potentially include glycan-mediated contacts.

animals or cell cultures such as macrophages (Olejnik et al., 2017). In Reston GP, both the IFL and the TACE cleavage sites are highly conserved. Thus, it is likely that the observed lack of cytokine production is due to an indirect effect. Reston has different glycosylation patterns where glycans are more extended and more prominent compared with other Ebola GPs, even when expressed under identical conditions. There are some distinct sequence differences that could possibly alter the glycosylation around the glycan cap and the N terminus but are also in proximity to the TACE cleavage site, which may affect enzyme activity through sterical shielding (Fujihira et al., 2018; Pappalardo et al., 2016). Alternatively, Reston GP has a mutation at the cleavage site required for processing into GP1 and GP2 and may be a poor substrate for Furin protease (Volchkov et al., 1998). Inefficient processing of GP correlates with reduced pathogenicity. Another possibility is that Reston GP forms a more stable trimer that does not dissociate, leading to lower TLR4 activity. The difference in pathogenicity between REBOV and Zaire EBOV (ZEBOV) cannot be directly linked to the activation of TLR4 but may be due to inefficient or impaired ability to infect human cells. It may be the case that REBOV is capable of activating TLR4; however, as the high levels of sGP1/2, which are required to trigger the severe cytokine storm, cannot be achieved with a low rate of infection, this will most likely go undetected.

In conclusion, we identify the EBOV IFL as a direct and potent activator of inflammatory signaling through TLR4. Further studies will elucidate the structure of TLR4/MD2 in complexes with sGP. Docking analysis suggests that GP can interact with both MD2 and TLR4 ECDs to stabilize the active hetero-tetrameric form. This also suggests that TLR4 antagonists such as eritoran (Younan et al., 2017) and TAK242 (Matsunaga et al., 2011) may be effective inhibitors of cytokine storm in late-stage Ebola infection and VHF. Structural analysis will also allow the development of inhibitors that are specific for Ebola sGP and potentially other viruses with IFLs that activate TLR4.

Limitations of the study

Although extensive mutagenesis was carried out of residues modified by N-linked glycosylation, two sites, N563 and N618, were refractory to analysis as the changes cause poor expression and defective trimer assembly. In addition, it was not possible to carry out a systematic study of O-linked glycans, which are primarily in the unstructured mucin domain and are thought to shield sGP1,2 from recognition by the immune system (Iraqi et al., 2020). Therefore, it is possible that glycosylation has an undefined secondary role in Ebola-induced inflammation. Protein-protein docking methods have improved greatly in the last 5 years, but it remains possible that the interactions observed do not or only partially recapitulate the interfaces formed *in vivo*.

STAR★METHODS

Detailed methods are provided in the online version of this paper and include the following:

- KEY RESOURCES TABLE
- RESOURCE AVAILABILITY
  - Lead contact
  - Materials availability
  - Data and code availability
- EXPERIMENTAL MODEL AND SUBJECT DETAILS
  - Cell culture

- METHOD DETAILS
  - Cloning and constructs
  - Protein expression
  - Protein purification
  - NFκB reporter assays
  - Immunoprecipitation
  - Protein de-glycosylation
  - Inhibition assays using *Rhodobacter sphaeroides* lipid A
  - TACE inhibition
  - Protein-protein docking
- QUANTIFICATION AND STATISTICAL ANALYSIS

SUPPLEMENTAL INFORMATION

Supplemental information can be found online at https://doi.org/10.1016/j.celrep.2022.111562.

ACKNOWLEDGMENTS

This work was funded by a Wellcome Trust Investigator award WT100321/z/12/Z to N.J.G. M.J.S. was supported by a studentship from the Oliver Gatty Fund for research on colloids (Cambridge University).

AUTHOR CONTRIBUTIONS

M.J.S. designed experiments, analyzed data, and interpreted results. M.G. analyzed data and interpreted results. N.J.G. analyzed data and wrote the paper.

DECLARATION OF INTERESTS

The authors declare no competing interests.

INCLUSION AND DIVERSITY

We support inclusive, diverse, and equitable conduct of research.

Received: March 28, 2022
Revised: September 16, 2022
Accepted: October 4, 2022
Published: October 25, 2022

REFERENCES

Akey, D.L., Brown, W.C., Dutta, S., Konwerski, J., Jose, J., Jurkiw, T.J., DelProposto, J., Ogata, C.M., Skiniotis, G., Kuhn, R.J., and Smith, J.L. (2014). Flavivirus NS1 structures reveal surfaces for associations with membranes and the immune system. Science *343*, 881–885.

Baseler, L., Chertow, D.S., Johnson, K.M., Feldmann, H., and Morens, D.M. (2017). The pathogenesis of ebola virus disease. Annu. Rev. Pathol. *12*, 387–418.

Breman, J.G., Heymann, D.L., Lloyd, G., McCormick, J.B., Miatudila, M., Murphy, F.A., Muyembé-Tamfun, J.J., Piot, P., Ruppol, J.F., Sureau, P., et al. (2016). Discovery and description of ebola Zaire virus in 1976 and relevance to the west African epidemic during 2013-2016. J. Infect. Dis. *214*, S93–S101.

Bryant, C.E., Gay, N.J., Heymans, S., Sacre, S., Schaefer, L., and Midwood, K.S. (2015). Advances in Toll-like receptor biology: modes of activation by diverse stimuli. Crit. Rev. Biochem. Mol. Biol. *50*, 359–379.

Bryant, C.E., Spring, D.R., Gangloff, M., and Gay, N.J. (2010). The molecular basis of the host response to lipopolysaccharide. Nat. Rev. Microbiol. *8*, 8–14.

Chen, R., Li, L., and Weng, Z. (2003). ZDOCK: an initial-stage protein-docking algorithm. Proteins *52*, 80–87.

Davidson, E., Bryan, C., Fong, R.H., Barnes, T., Pfaff, J.M., Mabila, M., Rucker, J.B., and Doranz, B.J. (2015). Mechanism of binding to ebola virus glycoprotein by the ZMapp, ZMAb, and MB-003 cocktail antibodies. J. Virol. *89*, 10982–10992.

Dolnik, O., Volchkova, V., Garten, W., Carbonnelle, C., Becker, S., Kahnt, J., Ströher, U., Klenk, H.D., and Volchkov, V. (2004). Ectodomain shedding of the glycoprotein GP of Ebola virus. EMBO J. *23*, 2175–2184.

Escudero-Pérez, B., Volchkova, V.A., Dolnik, O., Lawrence, P., and Volchkov, V.E. (2014). Shed GP of ebola virus triggers immune activation and increased vascular permeability. PLoS Pathog. *10*, e1004509.

Falasca, L., Agrati, C., Petrosillo, N., Di Caro, A., Capobianchi, M.R., Ippolito, G., and Piacentini, M. (2015). Molecular mechanisms of Ebola virus pathogenesis: focus on cell death. Cell Death Differ. *22*, 1250–1259.

Feldmann, H., Klenk, H.D., and Sanchez, A. (1993). Molecular biology and evolution of filoviruses. Arch. Virol. Suppl. *7*, 81–100.

Feldmann, H., Nichol, S.T., Klenk, H.D., Peters, C.J., and Sanchez, A. (1994). Characterization of filoviruses based on differences in structure and antigenicity of the virion glycoprotein. Virology *199*, 469–473.

Fujihira, H., Usami, K., Matsuno, K., Takeuchi, H., Denda-Nagai, K., Furukawa, J.I., Shinohara, Y., Takada, A., Kawaoka, Y., and Irimura, T. (2018). A critical domain of ebolavirus envelope glycoprotein determines glycoform and infectivity. Sci. Rep. *8*, 5495.

Gooz, M. (2010). ADAM-17: the enzyme that does it all. Crit. Rev. Biochem. Mol. Biol. *45*, 146–169.

Gregory, S.M., Harada, E., Liang, B., Delos, S.E., White, J.M., and Tamm, L.K. (2011). Structure and function of the complete internal fusion loop from Ebolavirus glycoprotein 2. Proc. Natl. Acad. Sci. USA *108*, 11211–11216.

Gupta, R., and Brunak, S. (2002). Prediction of glycosylation across the human proteome and the correlation to protein function. Pac. Symp. Biocomput., 310–322.

Iraqi, M., Edri, A., Greenshpan, Y., Kundu, K., Bolel, P., Cahana, A., Ottolenghi, A., Gazit, R., Lobel, L., Braiman, A., and Porgador, A. (2020). N-glycans mediate the ebola virus-GP1 shielding of ligands to immune receptors and immune evasion. Front. Cell. Infect. Microbiol. *10*, 48.

Jeffers, S.A., Sanders, D.A., and Sanchez, A. (2002). Covalent modifications of the ebola virus glycoprotein. J. Virol. *76*, 12463–12472.

Lee, J.E., Fusco, M.L., Hessell, A.J., Oswald, W.B., Burton, D.R., and Saphire, E.O. (2008). Structure of the Ebola virus glycoprotein bound to an antibody from a human survivor. Nature *454*, 177–182.

Lee, J.E., and Saphire, E.O. (2009). Ebolavirus glycoprotein structure and mechanism of entry. Future Virol. *4*, 621–635.

Leligdowicz, A., Fischer, W.A., 2nd, Uyeki, T.M., Fletcher, T.E., Adhikari, N.K.J., Portella, G., Lamontagne, F., Clement, C., Jacob, S.T., Rubinson, L., et al. (2016). Ebola virus disease and critical illness. Crit. Care *20*, 217.

Leroy, E.M., Kumulungui, B., Pourrut, X., Rouquet, P., Hassanin, A., Yaba, P., Délicat, A., Paweska, J.T., Gonzalez, J.P., and Swanepoel, R. (2005). Fruit bats as reservoirs of Ebola virus. Nature *438*, 575–576.

Lonez, C., Irvine, K.L., Pizzuto, M., Schmidt, B.I., Gay, N.J., Ruysschaert, J.M., Gangloff, M., and Bryant, C.E. (2015). Critical residues involved in Toll-like receptor 4 activation by cationic lipid nanocarriers are not located at the lipopolysaccharide-binding interface. Cell. Mol. Life Sci. *72*, 3971–3982.

Manček-Keber, M., Frank-Bertoncelj, M., Hafner-Bratkovič, I., Smole, A., Zorko, M., Pirher, N., Hayer, S., Kralj-Iglič, V., Rozman, B., Ilc, N., et al. (2015). Toll-like receptor 4 senses oxidative stress mediated by the oxidation of phospholipids in extracellular vesicles. Sci. Signal. *8*, ra60.

Matsunaga, N., Tsuchimori, N., Matsumoto, T., and Ii, M. (2011). TAK-242 (resatorvid), a small-molecule inhibitor of Toll-like receptor (TLR) 4 signaling, binds selectively to TLR4 and interferes with interactions between TLR4 and its adaptor molecules. Mol. Pharmacol. *79*, 34–41.

Misasi, J., and Sullivan, N.J. (2014). Camouflage and misdirection: the full-on assault of ebola virus disease. Cell *159*, 477–486.

CellPress
OPEN ACCESS

Stacey, K.J., Watterson, D., Modhiran, N., Young, P.R., Sester, D.P., Liu, L., Hume, D.A., Stacey, K.J., and Young, P.R. (2015). Dengue virus NS1 protein activates cells via Toll-like receptor 4 and disrupts endothelial cell monolayer integrity. Sci. Transl. Med. *7*, 304ra142.

Moss, M.L., and Minond, D. (2017). Recent advances in ADAM17 research: a promising target for cancer and inflammation. Mediators Inflamm. *2017*, 9673537.

Okumura, A., Pitha, P.M., Yoshimura, A., and Harty, R.N. (2010). Interaction between Ebola virus glycoprotein and host toll-like receptor 4 leads to induction of proinflammatory cytokines and SOCS1. J. Virol. *84*, 27–33.

Olejnik, J., Forero, A., Deflubé, L.R., Hume, A.J., Manhart, W.A., Nishida, A., Marzi, A., Katze, M.G., Ebihara, H., Rasmussen, A.L., and Mühlberger, E. (2017). Ebolaviruses associated with differential pathogenicity induce distinct host responses in human macrophages. J. Virol. *91*, e00179-17.

Olejnik, J., Hume, A.J., and Mühlberger, E. (2018). Toll-like receptor 4 in acute viral infection: too much of a good thing. PLoS Pathog. *14*, e1007390.

Pappalardo, M., Juliá, M., Howard, M.J., Rossman, J.S., Michaelis, M., and Wass, M.N. (2016). Conserved differences in protein sequence determine the human pathogenicity of Ebolaviruses. Sci. Rep. *6*, 23743.

Park, B.S., Song, D.H., Kim, H.M., Choi, B.S., Lee, H., and Lee, J.O. (2009). The structural basis of lipopolysaccharide recognition by the TLR4-MD-2 complex. Nature *458*, 1191–1195.

Pettersen, E.F., Goddard, T.D., Huang, C.C., Couch, G.S., Greenblatt, D.M., Meng, E.C., and Ferrin, T.E. (2004). UCSF Chimera–a visualization system for exploratory research and analysis. J. Comput. Chem. *25*, 1605–1612.

Pierce, B.G., Hourai, Y., and Weng, Z. (2011). Accelerating protein docking in ZDOCK using an advanced 3D convolution library. PLoS One *6*, e24657.

Pizzuto, M., Lonez, C., Baroja-Mazo, A., Martínez-Banaclocha, H., Tourlomousis, P., Gangloff, M., Pelegrin, P., Ruysschaert, J.M., Gay, N.J., and Bryant, C.E. (2019). Saturation of acyl chains converts cardiolipin from an antagonist to an activator of Toll-like receptor-4. Cell. Mol. Life Sci. *76*, 3667–3678.

Pourrut, X., Kumulungui, B., Wittmann, T., Moussavou, G., Délicat, A., Yaba, P., Nkoghe, D., Gonzalez, J.P., and Leroy, E.M. (2005). The natural history of Ebola virus in Africa. Microbes Infect. *7*, 1005–1014.

Prohinar, P., Re, F., Widstrom, R., Zhang, D., Teghanemt, A., Weiss, J.P., and Gioannini, T.L. (2007). Specific high affinity interactions of monomeric Endotoxin{middle dot}Protein complexes with toll-like receptor 4 ectodomain. J. Biol. Chem. *282*, 1010–1017.

Raghavan, B., Martin, S.F., Esser, P.R., Goebeler, M., and Schmidt, M. (2012). Metal allergens nickel and cobalt facilitate TLR4 homodimerization independently of MD2. EMBO Rep. *13*, 1109–1115.

Rutten, L., Gilman, M.S.A., Blokland, S., Juraszek, J., McLellan, J.S., and Langedijk, J.P.M. (2020). Structure-based design of prefusion-stabilized filovirus glycoprotein trimers. Cell Rep. *30*, 4540–4550.e3.

Tovchigrechko, A., and Vakser, I.A. (2006). GRAMM-X public web server for protein-protein docking. Nucleic Acids Res. *34*, W310–W314.

Volchkov, V.E., Feldmann, H., Volchkova, V.A., and Klenk, H.D. (1998). Processing of the Ebola virus glycoprotein by the proprotein convertase furin. Proc. Natl. Acad. Sci. USA *95*, 5762–5767.

Wang, B., Wang, Y., Frabutt, D.A., Zhang, X., Yao, X., Hu, D., Zhang, Z., Liu, C., Zheng, S., Xiang, S.H., and Zheng, Y.H. (2017). Mechanistic understanding of N-glycosylation in Ebola virus glycoprotein maturation and function. J. Biol. Chem. *292*, 5860–5870.

Wang, H., Shi, Y., Song, J., Qi, J., Lu, G., Yan, J., and Gao, G.F. (2016a). Ebola viral glycoprotein bound to its endosomal receptor niemann-pick C1. Cell *164*, 258–268.

Wang, Y., Su, L., Morin, M.D., Jones, B.T., Whitby, L.R., Surakattula, M.M.R.P., Huang, H., Shi, H., Choi, J.H., Wang, K.W., et al. (2016b). TLR4/MD-2 activation by a synthetic agonist with no similarity to LPS. Proc. Natl. Acad. Sci. USA *113*, E884–E893.

Weissenhorn, W., Carfí, A., Lee, K.H., Skehel, J.J., and Wiley, D.C. (1998). Crystal structure of the Ebola virus membrane fusion subunit, GP2, from the envelope glycoprotein ectodomain. Mol. Cell *2*, 605–616.

Yan, Y., Tao, H., He, J., and Huang, S.Y. (2020). The HDOCK server for integrated protein-protein docking. Nat. Protoc. *15*, 1829–1852.

Younan, P., Ramanathan, P., Graber, J., Gusovsky, F., and Bukreyev, A. (2017). The toll-like receptor 4 antagonist eritoran protects mice from lethal filovirus challenge. mBio *8*, e00226-17.

Zimmer, S.M., Liu, J., Clayton, J.L., Stephens, D.S., and Snyder, J.P. (2008). Paclitaxel binding to human and murine MD-2. J. Biol. Chem. *283*, 27916–27926.

STAR★METHODS

KEY RESOURCES TABLE

REAGENT or RESOURCE	SOURCE	IDENTIFIER
Antibodies		
Mouse monoclonal anti-His	BD Pharmingen	Cat#51-9000012
Rabbit polyclonal anti-TLR4	Abcam	Cat#Ab13867
Rabbit polyclonal anti-MD2	Abcam	Cat#Ab24182
Mouse Monoclonal anti-Rabbit HRP	Sigma	Cat#SAB5600195
Rabbit Polyclonal anti-mouse HRP	Sigma	Cat#AP160
Bacterial and virus strains		
NEB 5-alpha Competent E. coli	NEB	Cat#C2987H
Chemicals, peptides, and recombinant proteins		
PNGase F	New England Biolabs	P0704S
Endo-H	New England Biolabs	P0702S
O-Glycosidase	New England Biolabs	P0733S
β1-3,4 Galactosidase	New England Biolabs	P0746S
α2-3,6,8,9 Neuraminidase A	New England Biolabs	P0722S
Trypsin, Bovine Pancreas	Sigma	Cat#6502-2.5MU
Pepsin, Porcine Stomach Mucosa	Sigma	Cat#516360-500MG
hMD2	Abcam	Cat#Ab238343
hTLR4	Abcam	Cat#Ab233665
LPS-EB	InvivoGen	Cat#tlrl-eblps
LPS-RS	InvivoGen	Cat#tlrl-prslps
InSolution TAPI-1-Calbiochem	Merck	Cat#579052
Protein A/G beads	Abcam	Cat#Ab193262
Critical commercial assays		
Dual-Glo® Luciferase Assay System	Promega	Cat#E2920
Experimental models: Cell lines		
HEK293T	ATCC	Cat#CRL-1573
293/hTLR4-MD2-CD14	InvivoGen	Cat#293-htlr4md2cd14
HEK293S GnTI-	ATCC	Cat#CRL-3022
Oligonucleotides		
Primer for cloning of EBOV GP constructs see Table S2	This paper	N/A
Software and algorithms		
Prism	Graphpad	https://www.graphpad.com/scientific-software/prism/

RESOURCE AVAILABILITY

Lead contact

Further information and requests for resources and reagents should be directed to and will be fulfilled by the lead contact, Nicholas J. Gay (njg11@cam.ac.uk).

Materials availability

Materials are available from the lead contact.

Data and code availability

- Signaling assay data reported in this paper will be shared by the lead contact upon request.
- This paper does not report original code.
- Any additional information required to reanalyze the data reported in this paper is available from the lead contact upon request.

EXPERIMENTAL MODEL AND SUBJECT DETAILS

Cell culture

HEK293T cells (Invitrogen, Thermo Scientific, female) were maintained in DMEM Media GlutaMAX with 10% heat inactivated FBS and 100 U/mL of penicillin/streptomycin solution at 37°C and 5% CO_2 (all from Gibco, Life Technologies). hTLR4-MD2-CD14 HEK293 cells were maintained in DMEM Media GlutaMAX supplemented with 10% heat inactivated FBS, 100 U/mL of penicillin/streptomycin, normocin (100 μg/mL), and the selective antibiotics blasticidin (10 μg/mL) for hTLR4 and Hygromycin B Gold (50 μg/mL) (InvivoGen). Expi293 GnTI- cells were maintained in DMEM: F-12 Medium (Gibco) supplemented with 10% heat inactivated FBS, 100 U/mL of penicillin/streptomycin and 10% PLURONIC F-68 (all from Gibco) at 140 rpm, 37°C and 5% CO2.

METHOD DETAILS

Cloning and constructs

GP constructs for expression in mammalian cells were generated using the sequence for ZEBOV GP Makona (GenBank: AAB81004.1) as a template and a synthetic gene was obtained from Synbio Technologies. The construct was designed to incorporate a Strep-II tag, poly-His tag and TEV cleavage sequence at the N-terminal between signaling peptide and the GP gene. Full length constructs of GP with an intact transmembrane domain were cloned into pcDNA3.1 (+) using NheI and NotI sites while constructs optimised for protein secretion were cloned into pDisplay-AP-CFP-TM using HindIII and BamHI sites. All cloning was conducted using the Q5 High-Fidelity DNA Polymerase (New England Biolabs) including site-directed mutagenesis for glycosylation mutants of GP according to manufacturer's instruction. The IFL GP mutant was created by deleting the IFL with the following primers (forward primer GAAGGAATTTACACAGAGG, reverse primer TTCATCCTGAGTAGTCCAG). Following the PCR according to the manufacturers protocol of Q5 High-Fidelity DNA Polymerase (New England Biolabs), blunt end ligation was performed with this devised protocol: Kinase treatment at 37°C for 30min, and inactivate 65°C for 20min (28μL of PCR product, 3μL T4 ligase buffer, 0.5μL T4 Polynucleotide Kinase), digestion of template at 37°C for 1h, and inactivate at 80°C for 20 min (to kinase mix add 3.6μL Cutsmart buffer, 1μL Dpn1) and the plasmid ligation at 16°C overnight followed by inactivation at 65°C for 10min (mix 50ng of Dpn1 digested mix and add 2μL T4 ligase buffer, 1μL T4 DNA ligase in 20μL final volume). All buffers and enzymes were provided from New England BiolabsThe constructs pRK5F-TACE (31713) and Furin-bio-His (51755) were obtained from Addgene, pBIIX-NF-κB-Luc and hRG-TK (Renilla luciferase) were gifted by Dr Lee Hopkins (Department of Vet Medicine, University of Cambridge), while constructs for TLR4, MD2 and CD14 were generated in house.

Protein expression

Transfection of HEK293 cells was performed with linear PEI (Merck) at a ratio of 1:5 DNA to PEI and incubated with the cells for 16 h. Post transfection the medium was replenished with 5x the recommended media volume. 5 days post transfection the media was harvested, filtered through SteriCup vacuum filters and concentrated using Vivaspin 20 centrifugal concentrator PES, 100kDa molecular weight cut-off (Sartorius) in presence of 100X protease inhibitor cocktail (Calbiochem) at 4°C. Prior to protein purification the concentrated medium solution was dialyzed overnight using Spectra/Por 3 dialysis membrane standard RC tubing with a molecular weight cut-off of 6 kDa (Spectrumlabs) in calibration buffer appropriate to the column.

Protein purification

The proteins expressed in HEK293 cells were purified in a three-step process: Nickel IMAC, Ion-exchange chromatography and size exclusion chromatography. Nickel IMAC was performed using a 5 mL HisTrap excel column with HisTrap buffer (20 mM HEPES, 100 mM NaCl, 40 mM Imidazole, pH 8.0) and eluted with HisTrap elution buffer (20 mM HEPES, 75 mM NaCl, 400 mM Imidazole, pH 8.0), followed by TEV Protease treatment for tag removal. Anion exchange chromatography was performed with a 5 mL HiTrap Q column with HiTrap buffer (20 mM HEPES, 100 mM NaCl, 100 mM Imidazole, pH 8.0) and eluted using HiTrap buffer (20 mM HEPES, 1 M NaCl, pH 8.0). For the final purification step the sample was diluted in SEC buffer (20 mM HEPES, 100 mM Tris, 500 mM NaCl, 1 mM EDTA, pH 8.0), concentrated and run on a Superdex 200 GL10/300 column. All columns were obtained from Cytivia.

NFκB reporter assays

hTLR4/MD2/CD14 stably expressing HEK293 cells were seeded on 96-well flat bottom plates at a concentration of 1.5×10^5 cells/mL 48 h prior to transfection. 16 h post-transfection with the vectors pBIIX-NF-κB-Luc and hRG-TK (Renilla luciferase), the medium was replaced with advanced serum-free DMEM GlutaMAXX medium (Gibco) containing the ligand of interest and incubated at 37°C for 6 h. Following the induction the cells were washed with RT sterile 1x PBS, 70 μL of passive lysis buffer (Promega) was added to each well and the plate was stored at – 80°C. The cell lysates were assayed for luciferase activity using the Dual-Glo luciferase kit (Promega) with a modified protocol. Of each sample 25 μL were taken and incubated with first 50 μL of luciferase substrate buffer II, followed by 50 μL of Stop Glo solution. Luminescence signals were detected and analyzed using PHERAstar FS (BMG Labtech) plate reader. The relative activity was obtained by normalizing the NF-κB promotor luciferase signal to constitutively active Renilla luciferase.

Immunoprecipitation

HEK293T cell expressing the proteins of interest in 6-well plates were washed with 1x PBS and lysed with 200 μL HEPES lysis buffer (50 mM HEPES, 150 mM NaCl, 2 mM EDTA, 10% glycerol, 0.5% NP-40 pH 7.5), supplemented 100X protease inhibitor cocktail (Calbiochem) at 4°C for 1 h and subsequent lysate clearance by centrifugation at 16,000 g at 4°C for 10 min. In case of immunoprecipitation of purified proteins, 2μg of each target protein, (5 μg of GP proteins) mixed with HEPES lysis buffer to a final volume of 20 μL. For cell-free immunoprecipitation purified GP, and commercially available pure MD2 (ab238343, Abcam) and TLR4 (ab233665, Abcam) were incubated with 2 μL anti-MD2 (ab24182, Abcam), anti-TLR4 (ab13867, Abcam) and anti-His (51-9000012, BD-Pharmagen) antibodies at 4°C for 4 h followed by addition of 20 μL protein A/G beads (Abcam) and further 2 h incubation. The beads were washed with HEPES lysis buffer and eluted with 1 M Glycine pH 3 followed by NaOH neutralization.

Protein de-glycosylation

For the complete removal of glycans of GP, the recommended protocol of the manufacturer (New England Biolabs) was adapted. Reaction volumes were increased to 500 μL and the amount of enzyme upscaled accordingly. Instead of denaturing and boiling protein samples, the deglycosylation mix was supplemented with 1.5 M Urea. Furthermore, the incubation time was extended to 16 h, 10% glycerol and protease inhibitor cocktail (CalBiochem) added to the mixture for stability. To stop enzymatic activity the samples were diluted in PBS and concentrated with Vivaspin centrifugal concentrator PES, 50kDa cut-off.

Inhibition assays using *Rhodobacter sphaeroides* lipid A

Ultrapure LPS from photosynthetic bacterium *Rhodobacter sphaeroides* (RSLA) was obtained from InvivoGen, dissolved in DMSO at a concentration of 1 mg/mL and stored at −20°C. Stable HEK293 cells expressing hTLR4/MD2/CD14, were cultured and transfected for luciferase signaling as previously described. Prior to the routine induction with TLR4 agonists for the luciferase assay, the cells were subjected to RSLA with dilutions from 100ng to 1 μg per well, and incubated for 1 h.

TACE inhibition

HEK293T cells and HEK293 cells stably expressing hTLR4/MD2/CD14 were cultured as previously described. The cells were seeded at 1.5×10^5 cells/mL on 96-well plate 48 h before transfection. HEK293T cells were transfected with full-length GP (with TMD) in pcDNA3.1 (+) and TACE in pRK5F, while stable HEK293 cells were transfected with NF-κB promotor luciferase and renilla luciferase. 24 h post-transfection the culture media was removed, the cells were washed with 1 x sterile PBS and incubated with 20 μL of TrypLE Express (Gibco) per well for 5 min. The individual cell cultures of GP and TACE expressing HEK293T and the stable HEK293 cells were merged and transferred to a new plate. To each well, 10 μL of 10 mM (500 μg/100 μL) InSolution TAPI-1-TACE inhibitor (Calbiochem) and 150 μL of DMEM medium were added to make up a final volume of 200 μL per well. The two cell lines were then co-cultured for 24 h at 37°C and harvested subsequently for analysis.

Protein-protein docking

The molecular details of human TLR4 and MD2 heterotetramer were extracted from the crystal structure of the LPS-ligated complex, PDB: 3FXI upon removal of the atomic coordinates of LPS (Park et al., 2009), while the structures of Ebola virus glycoprotein (EBOV GP) internal fusion loop (IFL) and secreted GP1,2 were derived from the trimeric prefusion in complex with a neutralizing antibody from a human survivor, PDB: 3CSY (Lee et al., 2008) and monomeric glycoprotein bound to its endosomal receptor Niemann-Pick C1, PDB: 5F1B (Wang et al., 2016a) respectively. Docking was performed with protein−protein docking engines GRAMM-X (Tovchigrechko and Vakser, 2006) and ZDOCK (Chen et al., 2003; Pierce et al., 2011). As a starting point, a series of blind protein−protein docking calculations were performed with IFL as a bait and TLR4-MD2 heterotetramer as a prey. Poses located at the dimer interface were then short-listed for targeted docking at binding hot spots using ZDOCK with GP1,2 monomers. Figures were generated using Chimera (Pettersen et al., 2004).

QUANTIFICATION AND STATISTICAL ANALYSIS

All experiments were repeated at least three times and the quantification of luciferase assays was based on quintuple samples from each repetition. The relative NF-κB fold change is presented as means of each normalised measurement and the indicated standard error. Statistical significance was analyzed by using a two-tailed ANOVA test, in which $p < 0.01$ was deemed significant.

Cell Reports, Volume *41*

Supplemental information

Activation of Toll-like receptor 4 by Ebola virus-shed glycoprotein is direct and requires the internal fusion loop but not glycosylation

Michael J. Scherm, Monique Gangloff, and Nicholas J. Gay

SUPPLEMENTAL INFORMATION

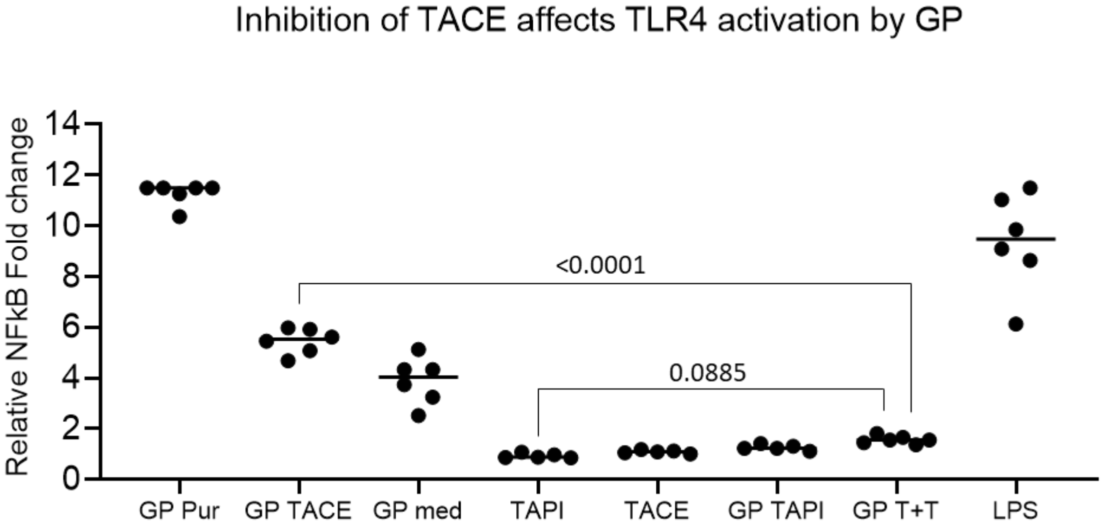

Figure S1: Inhibition of TACE eliminated GP-mediated TLR4 activation. (Related to Figure 2 and 3). The effect of inhibiting TACE activity on the secretion of GP and the activation of TLR4 was investigated. HEK293T cells were transfected with plasmids carrying full-length GP (including TMD) and/or TACE. Post-transfection induction the cells were washed and dissociated with TrypLE Express and merged with cultured Stable TMC HEK293 cells. The TACE inhibitor TAPI-1 was added to selected wells at 10 µM concentrations. The two cell lines were co-cultured for 24 hours and harvested for assessment of NFκB activity. 500 ng/µl of purified GP and 100ng/µl of LPS were used as positive controls. Expression of TACE alone, and transfection with an empty vector together with TAPI served as the negative control. The negative control was set to 1-fold change and the values normalised to it. Data points present individual replicates. Statistical significance was assessed using a one-way ANOVA test.

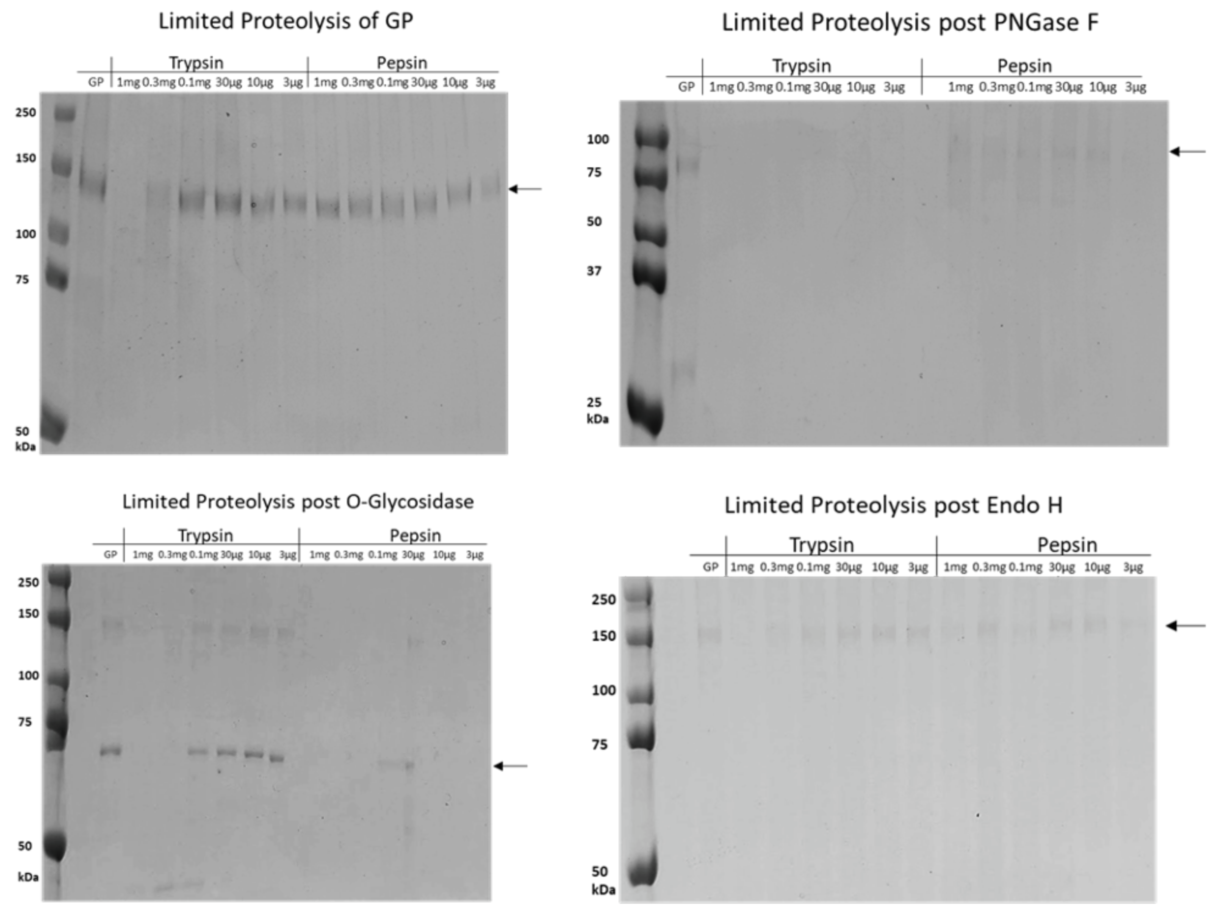

Figure S2: Partial proteolysis of GP post-deglycosylation with Trypsin and Pepsin. (Related to Figure 4). The impact of deglycosylation by PNGase F, O-glycosidase and Endo H on the stability of GP was analysed by utilising partial proteolysis by the proteases Trypsin and Pepsin, at pH 7.0 and pH 4.5 respectively. The GP samples treated with proteases under different concentrations were run on a gradient 6-16% SDS-PAGE under non-reducing conditions. The concentrations of the proteases used for each sample are indicated by the value and amount/per ml for each lane. The detailed protocol is listed in section 7. The arrow indicates GP.

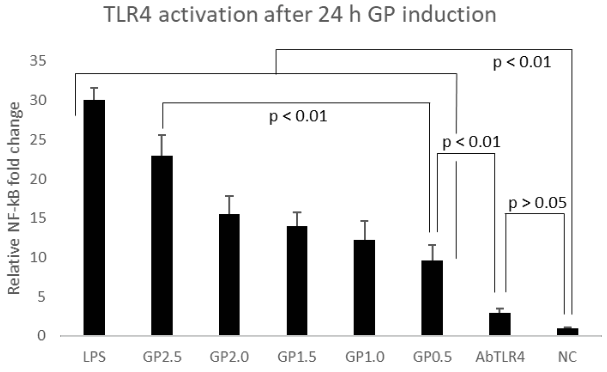

Figure S3 Dose-response curve for sGP activation of TLR4. ***(Related to Figure 5).*** NFκB activation was assayed at 5 different concentrations of sGP1,2 and in the presence of anti-TLR4 antibody.

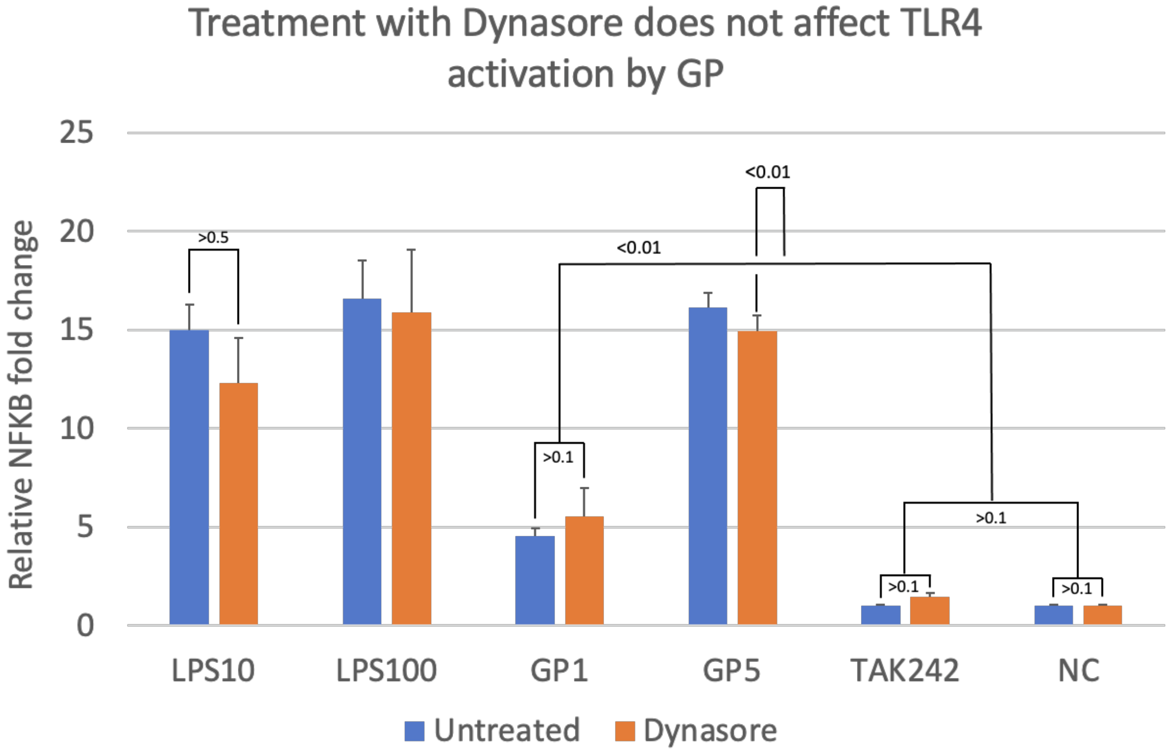

Figure S4 Treatment with Dynasore does not affect TLR4 activation by GP (related to Figure 5). Cells were either untreated (blue) or pre-treated with dynasore (80µM, orange) and then activated with the indicated ligands or the TLR4 inhibitor TAK242.

Position of Glycan	Sequence	Mutation	Expression
40	NSTL	N40D	No
40	NSTL	T42V	Yes
204	NATE	N204D	No
204	NATE	T206V	Yes
228	NETE	N228D	Yes
228	NETE	T230V	Yes
238	NLTY	N238D	Yes
238	NLTY	T240V	Yes
257	NETI	N257D	Yes
257	NETI	T259V	Yes
268	NTTG	N268D	No
268	NTTG	T270V	Yes
296	NLTR	N296D	No
296	NLTR	T298V	Yes
563	NETT	N563D	No
563	NETT	T565V	Yes
618	NITD	N618D	Yes
618	NITD	T620V	No

Table S1: Individual glycosylation site mutants of GP (related to Figure 4). Summary of the N-linked glycosylation sites of GP selected for mutant activity screen. The position and sequence of each site within the peptide chain is noted including the mutations of Asn to Glu and Thr to Val. The grey marked rows contain the mutants which did not express.

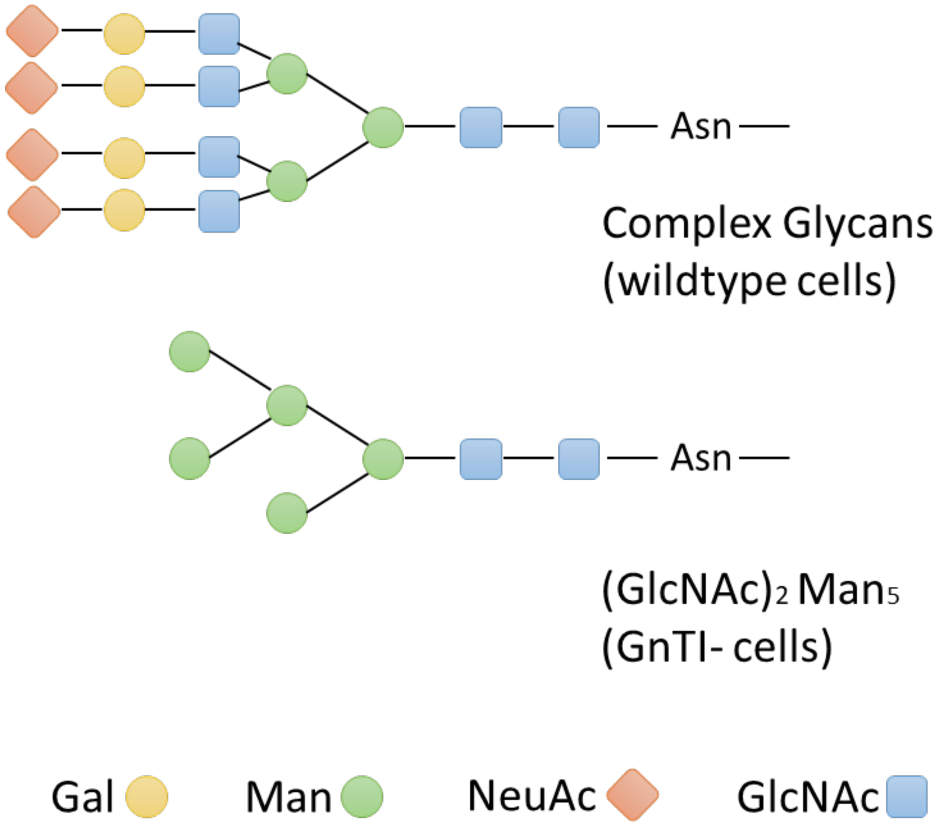

Figure S5: Schematic depiction of possible glycosylation in Expi293 GnTI- cells ***(related to Figure 4 and Figure 6).*** Comparison of glycosylation in HEK293T cells and GnTI- HEK cells. GnTI, N-acetylglucosaminyltransferase I; Gal, Galactose; Man, Mannose; NeuAc, N-Acetyl-Neuraminic Acid; GlcNAc, N-Acetylglucosamine; Asn, Asparagine.

Construct name	Forward primer	Reverse primer
GP pDisplay	ATATATAGGGCCCATCCCGCTTGGAGTTATC	ATTCTGTATATGCAAATTTGTCTTTTAGG GCGCGCCATATATAT
GP Synbio	GCCGCCAAGCTTGCGATCCCGCTTGGAGTTAT	GGCGGCAAGCTTCGCCTAAAAGACAAAT TTGCATATA
GP GP1	TAGGGCGCGCCGATGGTG	TCTTCGAGTCCTTCTCCCGCC
GP GP2	GAAGTAATTGTCAATGCTCAACCCAAATGCAACCCCA ATTTACATTACTGGACTACTCAG	GCTGCCGCCGCCGCCGTG
GPN40D	AGTTATCCACGATAGTACATTACAGGTTAGTGATGTC	CCAAGCGGGATGCTGCCG
GPT42V	CCACAATAGTGTATTACAGGTTAGTGATG	ATAACTCCAAGCGGGATG
GPN204D	AGAGCCGGTCGATGCAACGGAGG	CTCAAGGGGTGTGAGCTGAAG
GPT206V	GGTCAATGCAGTGGAGGACCCGTC	GGCTCTCTCAAGGGGTGT
GPN228D	TTTTGGAACTGATGAGACAGAGTAC	CCGGTAGCCTGATATCTAATTG
GPT230V	AACTAATGAGGTAGAGTACTTGTTCGAGGTTG	CCAAAACCGGTAGCCTGA
GPN238D	CGAGGTTGACGATTTGACCTACG	AACAAGTACTCTGTCTCATTAG
GPT240V	TGACAATTTGGTCTACGTCCAACTTG	ACCTCGAACAAGTACTCTG
GPN257D	GCTCCAGCTGGATGAGACAATAT	AGAAACTGTGGTGTGAATC
GPT259V	GCTGAATGAGGTAATATATGCAAGTGGG	TGGAGCAGAAACTGTGGT
GPN268D	GAAGAGGAGCGACACCACGGGAA	CCACTTGCATATATTGTCTCATTCAGC
GPT270V	GAGCAACACCGTGGGAAAACTAATTTG	CTCTTCCCACTTGCATATATTG
GPN296D	AACTAAAAAAGACCTCACTAGAAAAATTCGCAGTG	TCCCAGAAGGCCCACTCC
GPT298V	AAAAAACCTCGTTAGAAAAATTCGCAGTGAAG	TTAGTTTCCCAGAAGGCC
GPN563D	GCAGCTGGCCGACGAAACGACTC	CTCAACCCACAGATTAAACCATCTTGG
GPT565V	GGCCAACGAAGTGACTCAAGCTCTCCAAC	AGCTGCCTCAACCCACAG
GPN618D	TTGGACCAAGGACATAACAGACA	TCATGTGGTTCGATACAG
GPT620V	CAAGAACATAGTAGACAAAATTGATCAGATTATTC	GTCCAATCATGTGGTTCG
GPΔMLD	ACTCATCACCAAGATACC	TGATACAGCTGTGAAAGAC
GPΔTM	TGGTGGACAGGATGG	CACACTGGACTAGTGCCATCCTGTCCACC A
GP_MLD	ATGATGACCGGTGAACTGAGCTTCACCGT	CATCATGGTACCTGTGTTGGTGATCAGTC C
GP_485	ATGATGACCGGTATCCCCCTGGGCGTGAT	CATCATGGTACCTGTGTTGGTGATCAGTC CCAGC
GP_MLD_50 1	ATGATGACCGGTGAACTGAGCTTCACCGTGGTGT	CATCATGGTACCGCGTCTGGTTCTTCTGC CG
GP_ECD	CACGACTTCGTGGACTGAGGATCCCTGGG	GCCCAGGGATCCTCAGTCCACGAAGTCG T
GP_IFL	CTGGCCAACGAAACGACT	ATTGACAATTACTTCTCTTCGAGTC

Table S2: Primers used for EBOV GP cloning (related to Methods, Cloning and constructs). Summary of the oligonucleotides used to clone different mutants of EBOV GP. The names of each construct, the forward and the reverse primer are annotated.
